# Out of the forest: past and present range expansion of a parthenogenetic weevil pest, or how to colonize the world successfully

**DOI:** 10.1002/ece3.2180

**Published:** 2016-07-06

**Authors:** Marcela S. Rodriguero, Analía A. Lanteri, Noelia V. Guzmán, Jerson V. Carús Guedes, Viviana A. Confalonieri

**Affiliations:** ^1^ Departamento de EcologíaGenética y EvoluciónFacultad de Ciencias Exactas y NaturalesUniversidad de Buenos Aires, IEGEBA (CONICET‐UBA)Intendente Güiraldes y Costanera Norte s/n4to. Piso, Pabellón II, Ciudad UniversitariaCI1428 EHACiudad Autónoma de Buenos AiresArgentina; ^2^ División EntomologíaMuseo de La PlataFacultad de Ciencias Naturales y MuseoUniversidad Nacional de La PlataPaseo del Bosque s/n1900La PlataArgentina; ^3^ Departamento de Defesa FitossanitáriaCentro de Ciências RuraisUniversidade Federal de Santa MariaPrédio 42, Campus Universitário97105‐900Santa MariaRio Grande do SulBrazil

**Keywords:** Ecological niche modeling, invasive weevils, *Naupactus cervinus*, parthenogenesis, Pleistocene refugia, range expansion

## Abstract

Previous research revealed complex diversification patterns in the parthenogenetic weevil *Naupactus cervinus*. To understand the origin of clonal diversity and successful spreading of this weevil, we investigated its geographic origin and possible dispersal routes and whether parthenogens can persist in habitats under unsuitable environmental conditions. This study is based on samples taken throughout a broad area of the species’ range. We used both mitochondrial and nuclear markers and applied phylogenetic and network analyses to infer possible relationships between haplotypes. Bayesian phylogeographic analyses and ecological niche modeling were used to investigate the processes that shaped genetic diversity and enabled the colonization of new geographic areas. Southeastern Brazil emerges as the original distribution area of *N*. *cervinus*. We detected two range expansions, one along natural corridors during the Pleistocene and the other in countries outside South America during recent times. Isolation due to climate shifts during the early Pleistocene led to diversification in two divergent clades, which probably survived in different refugia of the Paranaense Forest and the Paraná River delta. The origin of the clonal diversity was probably a complex process including mutational diversification, hybridization, and secondary colonization. The establishment of *N*. *cervinus* in areas outside its native range may indicate adaptation to drier and cooler conditions. Parthenogenesis would be advantageous for the colonization of new environments by preventing the breakup of successful gene combinations. As in other insect pests, the present distribution of *N*. *cervinus* results from both its evolutionary history and its recent history related to human activities.

## Introduction

Asexual reproduction has long been claimed to be an evolutionary dead end due to the accumulation of deleterious mutations. However, some clonal species may be both as successful and persistent through evolutionary time as their sexual counterparts. A growing body of evidence supports the idea that some asexual lineages are actually quite ancient, such as rotifers of the class Bdelloidea (Waggonar and Poinar 1993), or may have a differential ability to settle in certain habitats (e.g., Mergeay et al. 2006). A better understanding of the apparent success of asexual organisms may help to unravel not only evolutionary questions (e.g., Why does sexual reproduction prevail despite its higher cost?), but also ecological ones, as those related to biological invasions.

The unexpected high level of diversity revealed by genetic analyses in many asexual systems (e.g., Cywinska and Hebert 2002; Shreve et al. 2011) has triggered research on the origin of clonal variation and on factors influencing its spatial distribution. Mechanisms of *in situ* diversification such as local polyphyly (i.e., interspecific hybridization, sex‐limited meiosis suppression and bacterial infection; Frati et al. 2004; Paland et al. 2005; Bi and Bogart 2006) or local divergence (i.e., mutation and also recombination in automictic parthenogens; Browne and Hoopes 1990) may lead to genetic diversity in clonal systems. Moreover, variation in local populations may also arise from a diverse pool of colonists (Cywinska and Hebert 2002). Regarding the ecological implications of clonal variation, co‐occurrence of clones is common (Weider et al. 1999), even though fitness differences were reported in most of asexual assemblies (Weider and Hebert 1987), in some cases linked to distributions in nature (Simon et al. 1999).

Disentangling whether genetic or ecological factors are prominent in shaping the patterns of clonal variation requires a historical perspective. In this regard, integration of both phylogeographic and ecological approaches can help understand the basis of the colonization process in parthenogenetic species.

The “Fuller's rose weevil” *Naupactus cervinus* Boheman (Curculionidae: Naupactini) is a very attractive model to investigate the evolution and consequences of asexual reproduction (e.g., Rodriguero et al. 2010b, 2013). It is a cosmopolitan species of economic importance native to South America that causes severe damages to ornamental plants, fruit trees and other crops (Guedes et al. 2005). Sexual populations of *N*. *cervinus* would have occurred until *c*. 70 years ago in the Paranaense Forest (Lanteri 1986, 1993), a humid subtropical forest in southeastern Brazil, eastern Paraguay, and northeastern Argentina, that reaches the La Plata River going down the gallery forests of the Paraná and Uruguay rivers. Currently, these gallery forests only harbor parthenogenetic lineages of *N*.* cervinus*, which also occur in grasslands and steppes of Argentina, Brazil, and Uruguay, and in other countries (Australia, Chile, Spain, USA, etc.) where it was introduced through commercial trade (Rodriguero et al. 2010b).

In previous contributions we demonstrated that *Naupactus cervinus* is a species complex with some divergent parthenogenetic lineages that are still undergoing speciation (Rodriguero et al. 2013). All lineages are infected with a single strain of *Wolbachia* (Rodriguero et al. 2010a,b, 2013), a bacterium that induces thelytokous parthenogenesis and other reproductive alterations in diverse arthropod hosts (Engelstädter and Hurst 2009). Moreover, we provided information concerning the nuclear and mitochondrial genetic diversity of the species across a large portion of its geographic range (Rodriguero et al. 2010a, 2013). We concluded that both parthenogenesis and *Wolbachia* infection have left an imprint on its genomes, such as coevolution between nucleus and mitochondria, indicating the ancient origin of asexual reproduction (Rodriguero et al. 2010b).

Asexuality provides demographic advantages for invading new areas. A single, unmated female can initiate a new colony, thus avoiding the impact of inbreeding depression on small founding populations and the cost of both male production and mate searching (Frankham 2005). On the other hand, asexual reproduction generally limits adaptive potential for colonizing new habitats, but preexisting adaptations and habitat tolerance of parthenogens are key factors contributing to a successful outcome. In this context, colonization most likely relies on genetically diverse founding populations or multiple introductions (Dlugosch and Parker 2008).

To unravel the origin of genetic variation in *N*. *cervinus*, the factors that shaped its spatial distribution, and the possible causes underlying its successful spread, we carried out a phylogeographic study coupled to an ecological niche modeling analysis to accurately recover the evolutionary history of this weevil.

## Material and Methods

### Biological material

**Figure 1:**
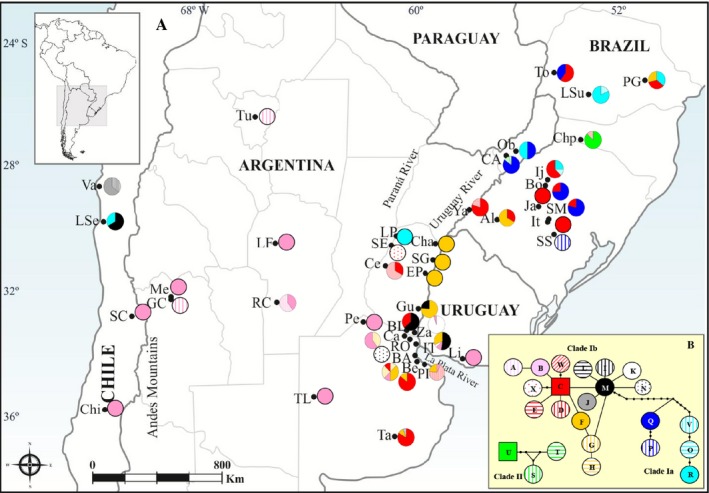
(A) Spatial distribution of mitochondrial genetic variation of *Naupactus cervinus* in South America. The pie chart at each sampling site shows relative frequencies of haplotypes. (B) Statistical parsimony network of mitochondrial haplotypes. Lines represent the most‐parsimonious relationships between haplotypes, open circles represent individual haplotypes, and unlabeled nodes indicate inferred steps not found in the samples. Rectangles indicate possible ancestral haplotypes. Circle size is not proportional to haplotype frequency. Clades Ia, Ib, and II refer to the haplotype groupings recovered by parsimony analysis in Figure 3. Loops indicate uncertain relationships between haplotypes.

**Figure 2:**
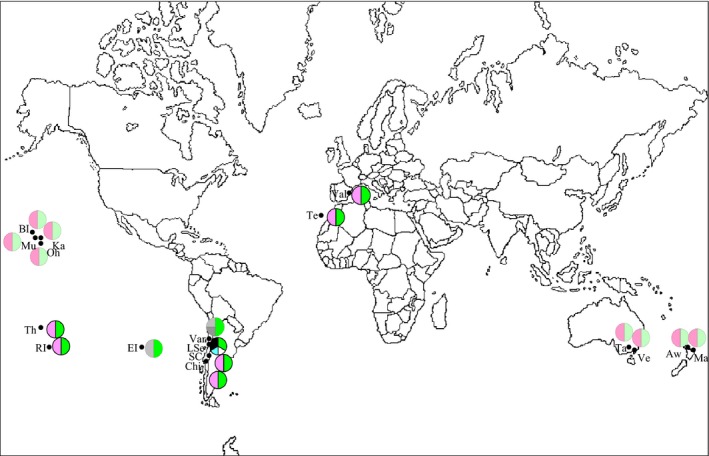
Worldwide spatial distribution of mitochondrial and nuclear genetic variation of *Naupactus cervinus*. The pie chart at each sampling site shows relative frequencies of haplotypes. The left and right side of the circle depicts the mitochondrial and nuclear variants, respectively.

Samples of adults of *N*.* cervinus* were collected across the species’ geographic range in South America and in non‐South American countries where it was likely recently introduced (Table 1; Figs. 1 and 2; *n *=* *417). Specimens were captured on wild and cultivated plants using a beating sheet, sexed under binocular microscope and stored at −80°C or in 100% ethanol at 4°C for molecular analysis.

**Table 1:** Geographic distribution and genetic diversity of *Naupactus cervinus* samples. Acronyms of sampling sites, latitude, longitude, sampling size, mitochondrial haplotypes, and nuclear alleles are specified for each location

Sampling location	Acronym	Latitude, Longitude	*n*	mtDNA haplotypes	nDNA alleles
AI, Canary Islands, Tenerife	Te	27**°** 27′ N, 16**°** 14′ W	5	B^a^	VII^a^
AR, Bs. As., Benavídez	Be	34° 24′ S, 58° 41′ W	7	C^b^ F^b^	VI^b^ ^e^, ^b^
AR, Bs. As., Buenos Aires City	BA	34**°** 36′ S, 58**°** 26′ W	8	B^a^ C^b^ F^a^ G^b^ H^a^	V^b^ VI^a^^,^ b VII^b^, ^e^, ^b^
AR, Bs. As., Cardales	Ca	34**°** 18′S, 58**°** 57′ W	5	B^a^ G^a^	VI^a^ VII^a^
AR, Bs. As., Reserva Otamendi	RO	34**°** 14′ S, 58**°** 52′ W	21	N^a^	VII^a^
AR, Bs. As., Pereyra Iraola	PI	34**°** 50′ S, 58**°** 8′ W	13	B^a^ D^a^ F^a^	VI^a^ VII^a^
AR, Bs. As., Pergamino	Pe	33**°** 54′ S, 60**°** 35′ W	2	B^a^	VII^a^
AR, Bs. As., Talavera Island	TI	34**°** 10′ S, 58**°** 30′ W	19	B^a^ F^a^ K^a^ M^a^	VI^a^ VII^a^
AR, Bs. As., Tandil	Ta	37**°** 19′ S, 59**°** 08′ W	18	B^a^ F^a^ L^a^	V^a^ VI^a^
AR, Bs. As., Tres Lomas	TL	36**°** 28′ S, 62**°** 52′ W	8	B^a^	VII^a^
AR, Bs. As., Zárate	Za	34**°** 06′ S, 59**°** 01′ W	20	B^a^ K^a^	VII^a^
AR, Córdoba, La Falda	LF	31**°** 05′ S, 64**°** 29′ W	13	B^a^	VII^a^
AR, Córdoba, Río Cuarto	RC	33**°** 08′ S, 64**°** 21′ W	5	A^a^ B^a^	VII^a^
AR, Corrientes, Yapeyú	Ya	29**°** 28′ S, 56**°** 50′ W	5	C^a^ E^a^	e, a
AR, E. Ríos, Brazo Largo	BL	33**°** 54′ S, 58**°** 53′ W	14	C^a^ M^a^	VII^a^
AR, E. Ríos, Cerrito	Ce	31**°** 34′ S, 60° 03′ W	3	C^b^ W^b^	V^b^ XVI^b^
AR, E. Ríos, Chajarí	Chj	30**°** 47′ S, 57**°** 59′ W	6	F^a^	VI^a^ VII^a^
AR, E. Ríos, El Palmar	EP	31**°** 50′ S, 58**°** 17′ W	14	F^a^	VI^a^
AR, E. Ríos, Gualeguaychú	Gu	33**°** 01′ S, 58**°** 31′ W	18	F^a^ M^a^	VI^a^ VII^a^
AR, E. Ríos, La Paz	LP	30**°** 45′ S, 59**°** 38′ W	6	R^b^	e, b
AR, E. Ríos, Salto Grande	SG	31**°** 23′ S, 58**°** 01′ W	12	F^a^	VI^a^
AR, E. Ríos, Santa Elena	SE	30**°** 56′ S, 59**°** 48′ W	2	X^b^	XVII^b^
AR, Mendoza, Godoy Cruz	GC	32**°** 56′ S, 68**°** 50′ W	3	A^a^	VII^a^
AR, Mendoza, Mendoza	Me	33**°** 30′ S, 69**°** 00′ W	3	B^a^	VII^a^
AR, Misiones, Cerro Azul	CA	27**°** 38′ S, 55**°** 30′ W	17	Q^a^ S^c^ T^c^	IV^a^ IX^c^ X^c^
AR, Misiones, Oberá	Ob	27**°** 29′ S, 55**°** 08′ W	2	Q^a^ R^a^	III^a^
AR, Tucumán, San Miguel de Tucumán	Tu	26**°** 46′ S, 65**°** 13′ W	1	A^b^	V^b^
AU, Victoria, Tatura	Ta	36**°** 26′S, 145**°** 13′ E	1	B^d^	V^d^
AU, Victoria, Vermont	Ve	37**°** 50′ S, 145**°** 11′ E	1	B^d^	V^d^
BR, PR, Laranjeiras do Sul	LS	25**°** 24′ S, 52**°** 24′ W	11	R^a^ V^b^	XII^b^ XIII^b^ XIV^b^ ^e^, ^a^
BR, PR, Ponta Grossa	PG	25**°** 05′ S, 50**°** 09′ W	14	C^a^ F^b^ R^b^	I^b^ VI^b^ ^e^, ^a^
BR, PR, Toledo	To	24**°** 42′ S, 53**°** 44′ W	10	C^a^ Q^b^	H^b^ ^e^, ^a^
BR, RG do Sul, Alegrete	Al	29**°** 46′ S, 55**°** 47′ W	6	C^a^ F^b^	VI^b^ VIII^b^ ^e^, ^a^
BR, RG do Sul, Bozano	Bo	28**°** 35′ S, 53**°** 59′ W	4	C^a^ Q^a^	II^a^
BR, RG do Sul, Ijui	Ij	28**°** 23′ S, 53**°** 54′ W	8	C^a^ O^b^ R^b^	XV^b^ ^e^, ^a^
BR, RG do Sul, Itaára	It	29**°** 36′ S, 53**°** 45′ W	9	C^a^	VIII^b^ ^e^, ^a^
BR, RG do Sul, Jari	Ja	29**°** 17′ S, 54**°** 13′ W	10	C^a^	XV^b^ ^e^, ^a^
BR, RG do Sul, Santa Maria	SM	29**°** 40′ S, 53**°** 47′ W	14	C^b^ Q^a^	IV^a^ ^e^, ^b^
BR, RG do Sul, São Sepé	SS	30**°** 10′ S, 53**°** 34′ W	11	P^a^	IV^a^
BR, SC, Chapecó	Chp	27**°** 03′ S, 52**°** 36′ W	8	E^a^ U^c^	e, a XI^c^
CH, Bío Bío, Chillan	Chi	36**°** 36′ S, 72**°** 06′ W	1	B^d^	VII^d^
CH, Region Metropolitana, Santiago	SC	33**°** 26′ S, 70**°** 29′ W	8	B^a^	VII^a^
CH, Coquimba, La Serena	LSe	29**°** 50′ S, 71**°** 14′ W	3	M^b^ R^b^	VII^b^ XVIII^b^
CH, Huasco, Vallenar	Var	28**°** 57′ S, 71**°** 15′ W	11	I^a^ J^a^	VII^a^
EU, Spain, Valencia	Val	39**°** 29′ N, 00**°** 23′ W	12	B^a^	VII^a^
NZ, Auckland, Awhitu	Aw	37**°** 05′ S, 174**°** 39′ E	1	B^d^	V^d^
NZ, Bay of Plenty, Matapihi	Ma	37° 41′ S, 176° 11′ E	1	B^d^	V^d^
PI, Easter Island (Rapa Nui)	EI	27° 08′ S, 109° 26′ W	7	I^a^	VII^a^
PI, French Polynesia, Rapa Island (Rapa Iti)	RI	27° 32′ S, 144° 20′ W	1	B^a^	VII^a^
PI, Hawaii, Big Island	BI	19° 36′ N, 155° 39′ W	1	B^b^	V^b^
PI, Hawaii, Kauai	Ka	22° 07′ N, 159° 31′ W	2	B^b^	V^b^
PI, Hawaii, Maui	Mu	20° 50′ N, 156° 20′ W	1	B^b^	V^b^
PI, Hawaii, Oahu	Oh	21° 28′ N, 157° 59′ W	1	B^d^	V^d^
PI, French Polynesia, Tahiti	Th	17° 52′ S, 149° 56′ W	3	B^a^	VII^a^
UR, Libertad	Li	34° 37′ S, 56° 37′ W	2	B^a^	VI^a^

AI, Atlantic Islands; AR, Argentina; AU, Australia; BR, Brazil; CH, Chile; EU, European Union; NZ, New Zealand; PI, Pacific Islands; UR, Uruguay.

Bs. As., Buenos Aires; E. Ríos, Entre Ríos; PR, Paraná; RS do Sul, Rio Grande do Sul; SC, Santa Catarina.

a Extracted from Rodriguero et al. (2010a).

b Obtained for the present work.

c Extracted from Rodriguero et al. (2013).

d Extracted from Mander et al. (2003).

e “Double peaks” individuals.

### PCR assay and sequencing

We extracted total genomic DNA following the Sunnucks and Hales’ protocol (Sunnucks and Hales 1996). The negative controls were samples lacking DNA template. A segment of *c*. 700 bp of the cytochrome *c* oxidase subunit I (*COI*) mitochondrial gene was amplified using the specific primers S1718 and A2442 (Rodriguero et al. 2010b, 2013). Additionally, a nuclear region of *c*. 1100 bp comprising the 3′ region of the 18S rDNA gene, the complete *ITS1* region (internal transcribed spacer 1) and the 5′ region of the 5.8S rDNA gene was amplified using the primers rDNA2 and rDNA 1.58S (Rodriguero et al. 2010b, 2013). Amplification was carried out under the conditions described by Rodriguero et al. (2010a).

DNA was sequenced using a 3130‐XL Automatic Sequencer (Applied Biosystems Foster City, CA).

### Data analysis

#### Genetic diversity and network construction

For comparison with previous contributions (Rodriguero et al. 2010b, 2013), phylogenetic analyses were performed by maximum parsimony using haplotypes as mitochondrial haplotypes or nuclear alleles as “terminal taxa” with TNT v. 1.1 (Goloboff et al. 2008). Gaps were treated as fifth state. Clade stability was assessed by 10,000 parsimony bootstrap replications. *Naupactus dissimulator* Boheman, the putative sister species of *N*. *cervinus* (Scataglini et al. 2005), was used as outgroup (Table S1). Additionally, a statistical parsimony analysis was conducted with all individual *COI* and *ITS1* sequences using the program TCS v. 1.21 (Clement et al. 2000) to generate haplotype and allele networks, respectively. The connection limit excluding homoplasic changes was set to 95%.

Estimation of nucleotide diversity was determined for each gene region and each clade by Watterson's (*θ*_*w*_), Tajima's (*π*), and haplotype diversity (Hd) estimators. All the calculations were performed with DnaSP v. 5.10.01 (Librado and Rozas 2009).

#### Phylogeographic analysis

We conducted a Bayesian phylogeographic analysis to infer the geographic origin of *N*. *cervinus* and changes in its distribution over time to infer when and where the ancestors of the studied species and populations existed, and reconstruct their phylogeographic history by applying a spatial diffusion approach (Bloomquist et al. 2010). We exclusively used the most variable dataset (i.e., mitochondrial sequences), because of incongruence due to incomplete lineage sorting of some *ITS1* sequences (Rodriguero et al. 2013). We used relaxed random walks applying the continuous diffusion (Bloomquist et al. 2010) following Lemey et al. (2010). The Cauchy RRW model was used for the continuous trait model prior (Lemey et al. 2010). Six independent MCMCs were run for 1000 million generations retaining samples every 100,000 generations, using beast v. 1.7.5 (Drummond and Rambaut 2007). Substitution models were selected using mrmodeltest software v. 2.2 (Nylander 2004) on the basis of the Akaike information criterion, and the GTR + I + G model was identified as the optimal model. We included only one sequence/haplotype/sampling site. When the same haplotype occurred at multiple sites, its frequency of occurrence is the number of sites where it was found. As some coordinates of the sampling localities were relatively close, we added random noise to identical or nearly identical coordinates using the jitter option with a parameter of 0.005. A “strict” clock model was assumed, based on the comparison of the likelihood scores of the trees obtained with the programs dnaml and dnamlk in the phylip software package v. 3.69 (Felsenstein 2005) through a likelihood ratio test (data not shown). The constant size was used as the tree prior and the starting tree was generated by UPGMA. The log‐likelihood scores of each run were checked for convergence of the chains in tracer v. 1.5 (Rambaut and Drummond 2007). Postburning trees from each run were combined with logcombiner v. 1.7.5 and summarized using treeannotator v. 1.7.5. A maximum clade credibility tree (MCC tree) with branch lengths representing posterior mean estimates was built with the remaining trees. We only analyzed ancestors that could be unambiguously reconstructed. Split and expansion times were estimated using a rate of 0.0177 substitutions/site/Myr (Papadopoulou et al. 2010) using *N*.* dissimulator* individuals from Argentina and Brazil as outgroup (see Table S1).

#### Ecological niche modeling

To estimate the most suitable environmental conditions and the potential distribution for *N*. *cervinus*, we modeled the probability distribution for its occurrence on the basis of several environmental constraints. This analysis is useful to test whether parthenogens are able to survive in unsuitable environments, especially those invading distant areas. The model was trained based on a set of 107 locations within the natural distribution of *N*. *cervinus* in South America (Table S2). To predict its current geographic distribution, we clipped the BIOCLIM rasters to a polygon containing the native range of *N*.* cervinus*, which was reasonably estimated; then, it was projected onto the world to show its potential distribution areas. To determine potential refugia during Pleistocene glaciations, we repeated this analysis for the Last Interglaciation (LIG; 120 ka) and the Last Glacial Maximum (LGM; 21 ka).

The probability distribution for the species’ occurrence was estimated with maxent v. 3.1 (Phillips et al. 2006). Then, we tested the different predictions with one of the occurrence records excluded in each prediction, using all records to generate the final potential habitat map. Taking into account the probability distribution for the occurrence of *N*. *cervinus*, we checked whether the species occupies unsuitable areas in the countries where it was recently introduced (127 locations; Table S3).

All bioclimatic variables available were tested for multicollinearity by examining cross‐correlations among variables (Pearson's correlation coefficient, *r*) in geographic space, based on occurrence records of *N*. *cervinus*. Only one variable from a set of highly correlated variables was included in the analysis (*r *≥* *0.80), based on its potential ecological influence on species’ distribution. This led us to select the following 11 environmental variables: Annual Mean Temperature; Mean Diurnal Range [mean of monthly (max temp − min temp)]; Isothermality; Max Temperature of Warmest Month; Min Temperature of Coldest Month; Annual Temperature Range; Mean Temperature of Wettest Quarter; Mean Temperature of Driest Quarter; Annual Precipitation; Precipitation of Wettest Month; and Precipitation of Driest Quarter. These were downloaded from worldclim 1.4 (http://www.worldclim.org/; accessed 16.01.2014) (Hijmans et al. 2005) and were combined and analyzed in a Geographic Information System. All layers were at 30‐arc‐sec (~1 km^2^) spatial resolution. In maxent we used the default convergence threshold (10^−5^) and increased maximum iterations to 1000. For each run, we used 75% of the localities to train the model and randomly selected 25% of the localities to test the model. We evaluated the model performance using a threshold‐independent method based on the area under the curve (AUC) of receiver operating characteristic curve (ROC) (Swets 1988). The contribution of each explanatory variable to model performance was evaluated with a jackknife procedure implemented in maxent. We transformed the output into a map representing probabilities of occurrence because maxent produces a continuous probability.

## Results

### Mitochondrial genetic diversity

All sampled individuals were females (*n = *417). The screening of mitochondrial genetic variation reveals 24 haplotypes from a sample of 395 sequences (Table 1). We also included sequences obtained by Mander et al. (2003) (*n = *6) from samples collected in Australia, Chile, Hawaii, and New Zealand, kindly provided by Dr Craig Phillips (Table 1).

Some haplotypes are restricted to single locations, whereas others occur at multiple sites (Figs. 1A and 2; Table 1).

**Figure 3:**
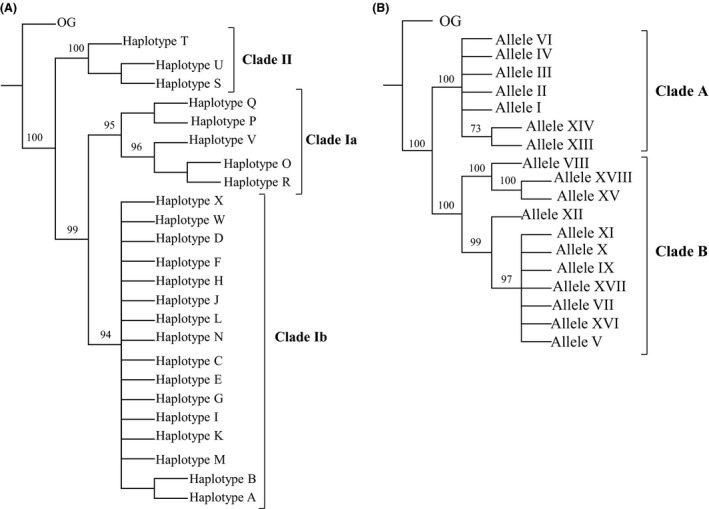
(A) Phylogenetic tree of COI haplotypes. Numbers above the branches are 50% or higher bootstrap values. Numbers above branches are ≥50% bootstrap values. (B) Phylogenetic tree of ITS1 alleles. Numbers above branches are ≥50% bootstrap values.

Maximum parsimony search yields 13 most‐parsimonious trees 138 steps long (Fig. 3A). We recovered three main highly supported clades, as in Rodriguero et al. (2010a, 2013). Clade Ia is composed of haplotypes O‐R and V, and it is mostly distributed over forest areas (northeastern Argentina and southeastern Brazil) (Fig. 1A). Clade Ib is composed of haplotypes A‐N, X, and W (Fig. 1A), and it is located on the banks of the Uruguay and Paraná rivers, at the confluence of the Paraná and Río de La Plata rivers and in the prairies of Argentina and Uruguay (Fig. 1A), as well as all over the world (Fig. 2; Table 1). Finally, clade II constitutes a separate lineage [probably a cryptic species according to Rodriguero et al. (2013)] and includes the haplotype S‐U, which co‐occurs in the forest area mentioned above (Fig. 1A).

Parsimony network displays three clades, in coincidence with results of the phylogenetic analysis (Fig. 1B).

Estimators of genetic variation for the whole sample and for every clade are shown in Table 2. Certainly, interclade divergence contributes to the overall values of *π* and *θ*_w_ for both markers. For the mitochondrial dataset, clade Ia seems to be more diverse than clades Ib and II (see *π* values), while the other estimators show similar values for the three groups.

**Table 2:** Genetic variation estimates of the mitochondrial and nuclear markers for the whole sample and each clade

Dataset	Clade	*N*	*π*	*θ*_*w*_	Hd
*COI*	Total sample	395	0.01134 ± 0.00074	0.01199 ± 0.00296	0.869 ± 0.00700
	Clade Ia	74	0.00624 ± 0.00034	0.00321 ± 0.00124	0.683 ± 0.00145
	Clade Ib	311	0.00270 ± 0.00008	0.00386 ± 0.00127	0.821 ± 0.00010
	Clade II	10	0.00321 ± 0.00101	0.00307 ± 0.00176	0.511 ± 0.16400
ITS1	Total sample	258	0.02274 ± 0.00075	0.01639 ± 0.00377	0.656 ± 0.00260
	Clade A	95	0.00213 ± 0.00023	0.00315 ± 0.00112	0.544 ± 0.03800
	Clade B	163	0.00570 ± 0.00131	0.01167 ± 0.00297	0.291 ± 0.04700

### Nuclear genetic diversity

Sequences of 326 individuals yielded a fragment of *c*. 1100 bp but only 258 individuals were eventually considered as the remainder had double‐peak chromatograms.

**Figure 4:**
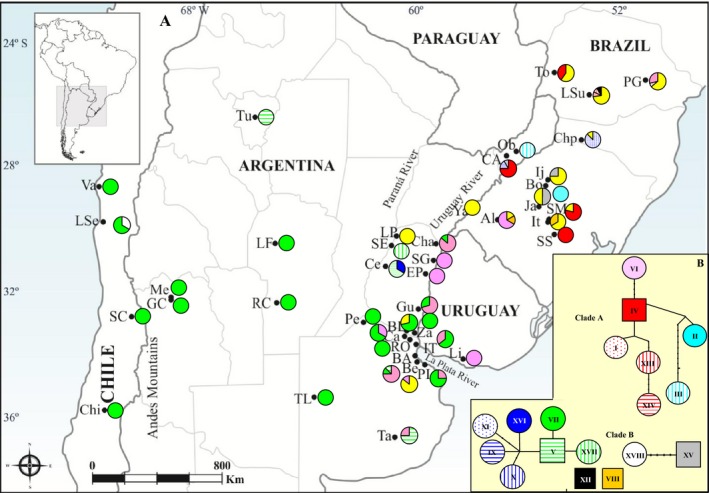
(A) Spatial distribution of nuclear genetic variation of *Naupactus cervinus*. The pie chart at each sampling site shows relative frequencies of alleles. (B) Statistical parsimony network of nuclear alleles. Lines represent the most‐parsimonious relationships between alleles, open circles represent individual alleles, and unlabeled nodes indicate inferred steps not found in the samples. Rectangles indicate possible ancestral alleles. Circle size is not proportional to allele frequency. Individuals yielding double‐peak chromatograms are shown in yellow. Clades A and B refer to haplotype groupings recovered by parsimony analysis in Figure 3.

Eighteen different alleles can be identified (Table 1). We also included the sequences obtained by Mander et al. (2003) (Table 1). The allele distribution pattern is similar to that in *COI* (Fig. 4A).

Maximum parsimony search yielded five most‐parsimonious trees 262 steps long (Fig. 3B). For this marker, only two divergent clades are recovered, namely clusters A and B (Rodriguero et al. 2013).

Statistical parsimony network identified five discrete lineages represented by five unlinked networks (Fig. 4B). In the clade A, alleles group together in a single lineage, with the allele IV occupying a central position. Another lineage has allele V occupying a central position in the network. It includes the alleles IX, X, and XI carried by individuals from clade II in the mitochondrial network. Finally, alleles VIII, XII, and the XV–XVIII group are in separate networks.

Genetic variation estimators for the whole sample and for every clade show that clade B (clade Ib + clade II) is much more diverse than clade A (clade Ia + clade Ib), although haplotypic diversity is lower in the first group.

### Phylogeographic analysis

**Figure 5:**
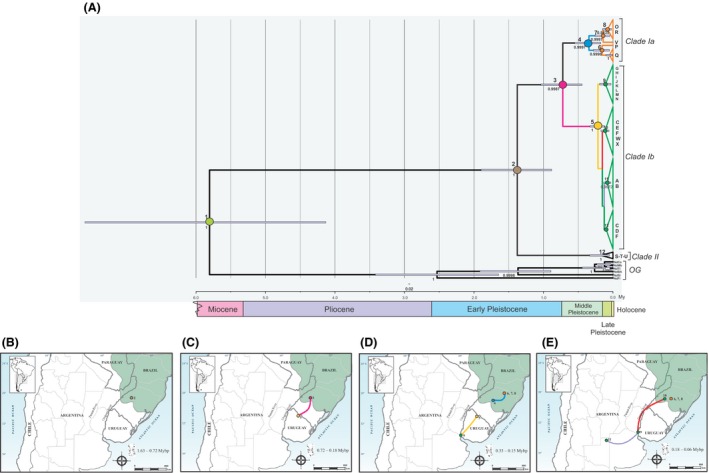
(A) Time‐calibrated maximum clade credibility tree from Bayesian phylogeographic analysis showing ancestors’ identity above branches and posterior probabilities >0.8 below branches. Numbers at the bottom indicate a relative time scale in millions of years before present. The scale bar represents the number of substitutions per site. The identity of every ancestor is used to portray the main events in the evolutionary history of *Naupactus cervinus* (see text for details). (B) Spatial distribution of mtDNA haplotypes of *Naupactus cervinus* over time (1.63–0.72 Myr). Distribution changes were inferred from a continuous phylogeographic analysis using a relaxed random walk. The timing of distribution changes are based on rates of sequence evolution in mtDNA following Papadopoulou et al. (2010), assuming a strict molecular clock. Scale bar is 800 km. The original surface of the Paranaense Forest is shown in green. (C) Spatial distribution of mtDNA haplotypes of *Naupactus cervinus* (between 0.72 and 0.18 Myr). (D) Spatial distribution of mtDNA haplotypes of *Naupactus cervinus* (between 0.33 and 0.15 Myr). (E) Spatial distribution of mtDNA haplotypes of *Naupactus cervinus* (between 0.18 and 0.01 Myr).

If the rate of molecular evolution in *COI* is consistent with that inferred for other insects, then *N*.* cervinus* probably split from its common ancestor with *N*. *dissimulator* at *c*. 5.8 Ma (95% HPD = 4.13–7.60 Ma; node 1, Fig. 5A). Bayesian phylogeographic inference supported a Brazilian origin for the ancestor of all the extant lineages of *N*. *cervinus* (northwestern Rio Grande do Sul state; node 2, Fig. 5A; brown dot, Fig. 5B). At some point between 0.72 (95% HPD = 0.41–1.00) and 0.18 Myr (95% HPD = 0.09–0.29), *N*. *cervinus* began to expand southward (node 3 and 5, Fig. 5A; pink line, pink and yellow dots, Fig. 5C). We call this demographic event “past expansion,” and it may have led *N*. *cervinus* to a continuous distribution over southeastern Brazil and Uruguay.

During this period, populations distributed in the northern limit differentiated from those in the south, giving rise to clades Ia and Ib (nodes 4 and 5, Fig. 5A; blue and yellow dots, Fig. 5D, respectively). Afterward, the two lineages continued to both diversify and expand (nodes 6, 7, 8, 9, 10, 11, and 12, Fig. 5A; blue and yellow lines and orange and green dots, Fig. 5D), and Ib reached the mouth of the Paraná River (known as “Paraná River delta” = PRD) (yellow line and green dot, Fig. 5D). Although the geographic location of the ancestors of most lineages belonging to the clade Ib was ambiguously reconstructed, the geographic distribution of its descendant haplotypes indicates an expansion from the PRD northward along the lower and middle reaches of the Uruguay and Paraná rivers (e.g., haplotypes C, E, F, W, and X, Fig. 1; node 10, Fig. 5A; red line, Fig. 5E) and eastward across the “Pampas” (e.g., haplotypes A and B, Fig. 1; node 11, Fig. 5A; purple line, Fig. 5E). These events may have occurred between 0.11 (95% HPD = 0.12–0.33) and 0.08 Myr (95% HPD = 0–0.10).

Finally, distant sampling locations are sites of human‐mediated introduction and we call it “present expansion.” Haplotypes I, J, M, and R may have colonized the Andean region (Fig. 1) and haplotype B, those countries outside South America like Australia, New Zealand, Spain, and some oceanic islands (Fig. 2).

### Niche modeling analysis and potential geographic distribution of *Naupactus cervinus*

**Figure 6:**
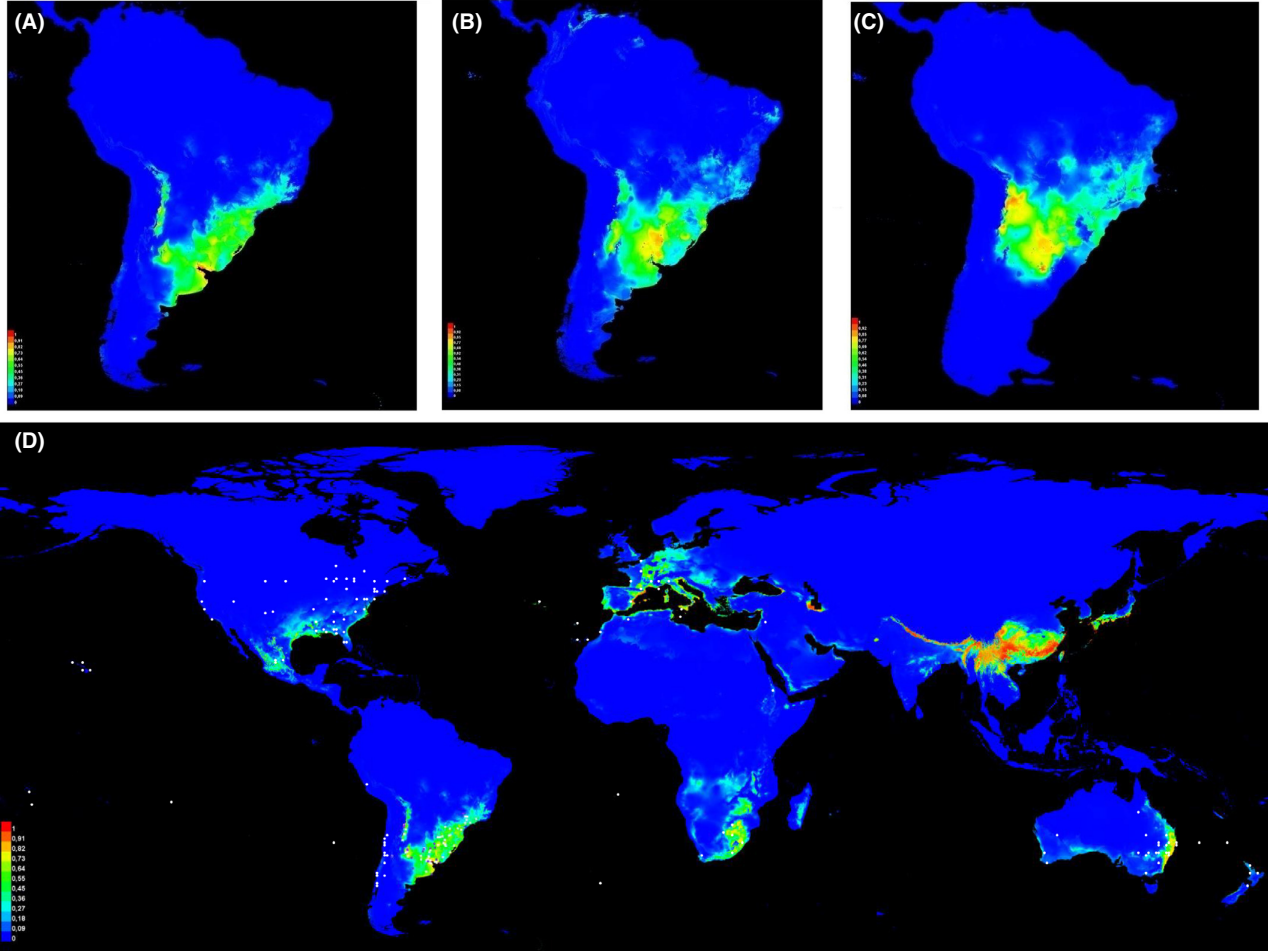
(A) Predicted geographic distribution of *Naupactus cervinus* based on current climatic conditions. Grid cells are classified by predicted suitability, with blue being least suitable and red being most suitable. (B) Predicted geographic distribution of *Naupactus cervinus* based on LIG climatic conditions. (C) Predicted geographic distribution of *Naupactus cervinus* based on LGM climatic conditions. (D) Predicted geographic distribution of *Naupactus cervinus* based on current climatic conditions projected onto the whole world. White dots correspond to localities outside South America where the species was recorded (see Table S3), which were not included in the niche modeling analysis.

We carried out ecological niche modeling to predict the area with the highest probability of *N*. *cervinus* occurrence in current times. Models performed better than random predictions (ROC AUC value = 0.962). The environmental variables with the highest training gain were annual mean temperature, mean temperature of coldest quarter, and mean temperature of wettest quarter. Overall, this analysis indicates that the most suitable areas for the occurrence of *N*. *cervinus* in South America are the confluence of the Paraná and Uruguay rivers and the Yungas rainforest, located in Bolivia and northwestern Argentina (*P *~ 0.90; Fig. 6A). Moderately suitable areas are found in southeastern Brazil, Uruguay, the Argentinean Mesopotamia (including the provinces of Misiones, Corrientes, and Entre Ríos), the prairies of central Argentina along the lower reach of the Uruguay River, and a narrow strip in the Paranaense Forest (0.55 < *P *<* *0.80; Fig. 6A).

Figure 6B shows the potential geographic distribution of *N*. *cervinus* predicted by maxent at *c*. 120 ka (LIG). Models performed better than random predictions (ROC AUC value = 0.991). Areas with highest probability of *N*. *cervinus* occurrence are larger compared to current suitable conditions; the former are mostly located in the Argentinean province of Entre Ríos, including the confluence of the Paraná and Uruguay rivers (0.85 < *P *<* *1.00; Fig. 6B). Moderately suitable areas also encompass prairies of central Argentina and a narrow strip in the Paranaense Forest (0.60 < *P *<* *0.70; Fig. 6B).

Figure 6C shows the potential geographic distribution of *N*. *cervinus* predicted by maxent at *c*. 21 ka (LGM). Again, models also performed better than random predictions (ROC AUC value = 0.962). Unexpectedly, the model predicts that *N*. *cervinus* has the highest probability of occurrence in a wider area than before. The most suitable area includes not only Entre Ríos but also Corrientes province, as well as small areas in northern Uruguay, western Rio Grande do Sul, and the Yungas (0.77 < *P *<* *0.92; Fig. 6C).

Finally, the projection of the model onto the world using current bioclimatic conditions also performed better than random predictions (ROC AUC value = 0.962). This analysis indicates that the highest probability of *N*. *cervinus* invasion is found in China, a small part of Iran, Georgia, Japan and the Mediterranean cost of Spain (*P *~ 1.0; Fig. 6D). Moderately suitable areas also encompass a narrow strip along the eastern coast of Australia, small patches in central Mexico and southeastern USA, a small region along the southeastern coast of Africa and Madagascar, and some patches on continental Europe (0.20 < *P *<* *0.70; Fig. 6D). However, the species has been reported as a serious pest in some areas where its probability of occurrence is almost nil (*P *~ 0.00; see white dots superimposed in Fig. 6D, from records of Table S3). Some examples are the west coast of Australia, central Chile, central USA, some patches in France, northern Africa, and New Zealand, and the islands of Juan Fernandez, Easter, Canary, and Polynesia.

## Discussion

### Past expansion: origin and spatial distribution of *Naupactus cervinus* genetic diversity

The study of the evolutionary history of *N*. *cervinus* suggests a range expansion during ancient and recent times from forest areas of southeastern Brazil to open vegetation areas.

Results of our phylogeographic analysis agree with Lanteri (1993), who proposed that the Paranaense Forest is the original distribution area of this weevil. This hypothesis is supported by the root location on the Bayesian tree and the higher mitochondrial genetic diversity found in this area. Coincidently, the closely related species *N*.* dissimulator* is also distributed in southeastern Brazil, northeastern Argentina, and Uruguay, showing highly divergent lineages in Brazil (A. A. Lanteri unpubl. ms.).

Dating results reveal an ancient origin for *N*. *cervinus* during the Pliocene and early Pleistocene, consistent with phylogeographic studies of species endemic to the Atlantic Forest (e.g., Carnaval et al. 2009). The origin and subsequent range expansion of the most derived lineages probably took place during late Pleistocene–Holocene.

The Paraná and Uruguay rivers serve as important biogeographic corridors connecting tropical and temperate biotas (e.g., Arzamendia and Giraudo 2009). The mesic microclimate on the riverbanks and the development of riparian humid forests known as “gallery forests” (Ringuelet 1961; Lutz 1986) may have facilitated the survival of tropical species such as *N*. *cervinus* and *N*. *dissimulator* at temperate latitudes when members of clade I spread southward.

The diversification of clade I into two main lineages (Ia and Ib) may have occurred by the presence of a barrier like those formed by the glaciations in the mid‐Pleistocene. The climatic changes in southern South America during this period are mostly explained by minor shifts in the location of the Pacific and Atlantic anticyclones (Antón and Goso 1974; Speranza et al. 2007). Several authors (e.g., Popolizio 1970; Antón and Goso 1974; Iriondo 1980; Zucol et al. 2004, 2005) assumed that there would have been a gradual predominance of the Pacific system, with a cooler, arid climate and steppe vegetation being followed by the alternation of semi‐arid temperate‐warm conditions (Atlantic system) and semi‐arid cooler conditions (Pacific system) in Entre Ríos province and Uruguay during the early‐mid‐Pleistocene. These climatic changes could have interrupted the cohesive forces that preserved clade I as a single evolutionary unit, thus favoring its diversification. An alternative hypothesis is that two parthenogenetic lineages originated independently. The hypothesis of a barrier disrupting the former continuous distribution range of *N*. *cervinus* appears as a more likely explanation, supported by distributions of other taxa like frogs and marsh deers showing a pattern of strong differentiation between populations of the PRD and Brazil (e.g., Williams and Bosso 1994; Márquez et al. 2006).

During these cold–dry phases, some lowland areas of Argentina and southeastern Brazil may have served as refugia for floras and faunas adapted to more humid climates (see Vuilleumier 1971). The Paranaense Forest possibly acted as a first refuge for *N*. *cervinus*, where it accumulated high levels of genetic diversity. Other refugia in this or nearby areas have been proposed (Ledru et al. 2007; Speranza et al. 2007; Werneck et al. 2011; Porto et al. 2013).

The PRD would be regarded as a second refuge for *N*. *cervinus*, where clade Ib would have reached a high level of genetic diversity. Further support to this hypothesis is provided by ecological niche modeling based on paleo‐environmental data indicating that *N*. *cervinus* has a high probability of survival in the PRD and the Entre Ríos province. This geographic area has also been proposed as a refuge for the marsh deer (Márquez et al. 2006).

The current distribution of the genetic variants in *N*. *cervinus* may be the result of a secondary contact among the formerly isolated lineages Ia, Ib (e.g., C and F mitochondrial haplotypes in Paraná and Rio Grande do Sul; Al and PG in Fig. 1) and II (e.g., haplotypes S, T, and U in Misiones and Santa Catarina; see Rodriguero et al. 2013) (green and orange dots in Fig. 5E, respectively). Surprisingly, Ib individuals may have migrated northward at *c*. 0.11–0.08 Myr, whereas Ia individuals seemed to have rarely ventured out of the relictual Paranaense Forest. Kröhling (2009) inferred temperate‐warm and humid climatic conditions for the late Pleistocene (0.088–0.080 Myr), during which paleo‐communities of subtropical to tropical flora were likely to co‐occur with some elements of the gallery forests (Brea and Zucol 2007; Brea et al. 2010). Moreover, Erra et al. (2013) assumed that the Entre Ríos province had a temperate‐warm climate during the LGM, with moisture increasing with time (see also Quattrocchio et al. 2008; Tonello and Prieto 2010). These conditions may have favored the northward migration of *N*. *cervinus* along the riparian forests of the Uruguay and Paraná rivers. Our results of paleoclimate modeling agree with these presumptions because we retrieved a larger suitable area for the *N*. *cervinus* during the LIG than at present (Fig. 6A vs. B). The appropriate environmental conditions persisted until LGM events, constituting an explanation for *N*.* cervinus* coming back home. Both Ia and Ib lineages may have coexisted for the last tens of thousands years. Another important result of our phylogeographic analysis is that individuals from clade Ib (haplotypes I, J, and M) colonized the areas along the Andean Cordillera, with some degree of diversification between 0.10 and 0.04 Myr. Indeed, our paleoclimate model indicates that a new suitable geographic area in northwestern Argentina could have favored the colonization of the vicinity of the Andes region during the LGM.

Prior studies on *N*. *cervinus* have shown remarkably high levels of clonal diversity (Rodriguero et al. 2010b, 2013; for comparison with other species, see table 4 in Shreve et al. 2011). Patterns of clonal diversification can be blurred if several processes contribute to the generation of diversity. Based on the results of the present work, which enlarged both the study area and the sampling effort of *N*. *cervinus*, it is difficult to ascertain a definitive answer as it is probable that more than one process can account for this astonishing level of clonal variation.

Processes that promote diversification of clones imprint distinctive signatures in populations of asexual taxa, as Cywinska and Hebert (2002) comprehensively detailed. Local polyphyly implies shifts in the allelic arrays of sexual and asexual lineages. Generation of diversity after the loss of sex will be evident upon rapid diversification of the nuclear variation associated to a common mitochondrial haplotype if automixis is the source of this variation, while close similarity between nuclear and mitochondrial markers will suggest mutation as the force behind diversification. Alternatively, external recruitment will impact in the form of high genetic divergence among the clones of a given assembly, as a consequence of their independent evolution.

The long history of mito‐nuclear coevolution in *N*. *cervinus* (Rodriguero et al. 2010b) suggests mutational diversification as a main source of clonal variation in this species, as ancestral mitochondrial haplotypes (and all its derived lineages) evolved along with the same nuclear haplotype across evolutionary times.

The phylogenetic gap between clades Ia and Ib could reflect a case of local polyphyly (i.e., independent origin or parthenogenesis). The fact that this pattern is also observed in sexual taxa inhabiting the same geographic area (Williams and Bosso 1994; Márquez et al. 2006) supports a demographic scenario as the source of this high divergence instead of two independent transitions to asexuality. Nevertheless, some clones of hybrid origin were discovered in this and previous works (“double peaks individuals”; see Table 1 and Rodriguero et al. 2010b). What we can assert is that these hybridizations occurred after the split of clade I, as we found both Ia (mitochondrial haplotypes R and V) and Ib (mitochondrial haplotypes C and E) mothers (nuclear genotypes are currently under study). At the same time, Paranaense Forest is currently harboring highly divergent clones, even in local populations (e.g., It, PG, SM, To) as a consequence of the secondary contact mentioned above. This co‐occurrence of clones sounds interesting, as clade Ib seems to be prone to face environmental variations (forest and open vegetation areas), in contrast to clade Ia. Thus, we are facing a complex origin of clonal diversity in *N*. *cervinus* which deserve further study.

### Present expansion of *Naupactus cervinus*: are some genetic clones more adapted to invasion?

The fact that parthenogenetic organisms are all female should have a significant demographic advantage over sexual organisms in colonizing new areas, as they may double the rate of population growth (Kearney 2005). In addition, parthenogenesis may promote establishment of coadapted gene complexes (Crow and Kimura 1965), which may facilitate ecological specialization (Sunnucks et al. 1997).

Many parthenogenetic species within Naupactini underwent geographic range expansions and exhibit higher colonization ability than sexual species (Lanteri and Normark 1995; Guzmán et al. 2012; Lanteri et al. 2013). However, it is worthwhile to mention that they established in suitable or moderately suitable environments (Guzmán et al. 2012; Lanteri et al. 2013). Evidence of a selective effect on the geographic range expansion in *N*. *cervinus* is provided by the remarkably high frequency of the B‐VII genotype, the only one present in a diversity of habitats having a low to null probability of occurrence (e.g., Canary Islands, Polynesia).

We already demonstrated linkage disequilibrium between nuclear and mitochondrial genomes for *N*. *cervinus* as a consequence of both asexuality and *Wolbachia* infection (Rodriguero et al. 2010b). For example, the mitochondrial haplotype B is associated with the nuclear allele VII, mainly in the “Pampas” (central Argentina). This genetic combination (and its derivative B‐V, differentiated by only one mutation), could be linked to the genetic variants involved in higher colonization ability, as suggested by the extraordinarily high incidence of the B‐VII genotype in the whole sample. Stenberg et al. (1997) proposed a similar explanation for the successful colonization of northern Europe by a single haplotype of the weevil *Otiorhynchus scaber*.

In turn, this coadapted gene complex would have enabled weevils to colonize countries outside South America through the commercial trade. Thus, we hypothesize that the B‐V genotype (and the emerging adaptations that may favor the ability to colonize adverse environments linked to these markers) was introduced at least once in Oceania, that the B‐VII genotype was introduced in Chile, Europe, and French Polynesia, and that the I‐VII, J‐VII, M‐VII, and R‐XVIII genotypes were introduced at least once in Chile.

Briefly, there may be factors other than parthenogenesis (and the demographic advantages associated to this reproductive mode) involved in the geographic range expansion of *N*. *cervinus*. However, lack of sex may have preserved those beneficial allele combinations being essential ingredients of a recipe for success in colonization of adverse environments. Parthenogenesis is assumed to have detrimental consequences in the long term, but in the short term it is paramount to the successful establishment of *N*. *cervinus* worldwide by preventing the breakup of coadapted gene complexes.

## Final Considerations

Our spatial analysis of genetic variation in *N*. *cervinus* from a phylogeographic approach suggests that it experienced two expansion waves: a past expansion into southern areas of open vegetation during the early‐mid‐Pleistocene, and a recent expansion into marginal areas outside South America. Climatic oscillations during the Ice Ages led to the interruption of the former continuous spatial distribution of this weevil across southeastern Brazil and the Argentinean Mesopotamia, giving rise to two divergent lineages, the clades Ia and Ib. Then, both populations came into secondary contact when climate shifted to the present‐day conditions.

Range expansion is a common pattern in the history of many species. Past climate fluctuations such those of the Pleistocene glacial–interglacial cycles modified species’ ranges considerably (Hewitt 2000). Phylogeographic studies may help understand the consequences of current global climate change affecting the range of species at an increasing rate. In particular, knowledge of the consequences of past events on the genetic variation distribution of *N*. *cervinus* has relevant implications for the conservation of the Paranaense Forest, as it is affected by accelerated biodiversity loss due to heavy anthropogenic pressure.

*Naupactus cervinus* has caused major economic losses in the countries where it was introduced. Our ecological niche modeling analysis indicates that special attention should be paid to prevent the introduction of *N*. *cervinus* through commercial foreign trade into China and the coast of the Caspian Sea, with optimal conditions for the colonization of this species. Additionally, natural enemies of *N*. *cervinus*, which share the original area (Rodriguero et al. 2014), should be thoroughly studied as an alternative to chemical pesticides. The considerations mentioned above emphasize the importance of knowing both *N*. *cervinus* original and potential distribution areas for the design of environmentally friendly control strategies.

## Conflict of Interest

The authors declare no conflict of interest.

## Data Accessibility

*COI* and *ITS1* sequences available from GenBank: GQ406827‐GQ406843, X440490, X440490, GU727685, and KX074095‐KX074098 for *COI* and GQ406818‐GQ406825, JX440499, KC614561, JX440500, and KX074088‐KX074094 for *ITS1*.

Details regarding sampling site localities and individual samples are available in Table 1.

## Supporting information


**Table S1.** Geographic distribution and genetic diversity of *Naupactus dissimulator* samples.

**Table S2.** Georeferenced localities for *Naupactus cervinus*, including literature records, examined material from entomological collections and field sampling used for molecular studies and ecological niche modeling.

**Table S3.** Georeferenced localities for *Naupactus cervinus*, including literature records, examined material from entomological collections and field sampling used for molecular studies and ecological niche modeling.
