# Supplementary material for: Out of the forest: past and present range expansion of a parthenogenetic weevil pest, or how to colonize the world successfully
Source: Ecol Evol. 2016 Jul 6;6(15):5431–45. doi: 10.1002/ece3.2180 (PMC4984515; doi:10.1002/ece3.2180)
Supplement: Supplementary file 1 — Table S1. Geographic distribution and genetic diversity of Naupactus dissimulator samples. [file ECE3-6-5431-s001.pdf]

Table S1 Geographic distribution and genetic diversity of *Naupactus dissimulator* samples. Acronyms of sampling sites, latitude, longitude, sampling size, mitochondrial haplotypes and nuclear alleles are specified for each location.

AR: Argentina; BR: Brazil; Bs. As.: Buenos Aires; E. Ríos: Entre Rios; SC: Santa Catarina.

Sampling location	Acronym	Lat / Long	N	mtDNA haplotypes	GenBank Accession No.
AR, Bs. As., Buenos Aires	BA	34º 36' S, 58º 26' W	1	C[a]	JX44049
AR, Bs. As., Talavera Island	IT	34º 10' S, 58º 30' W	1	A[a]	GQ406844
AR, Corrientes, Yapeyú	Ya	29º 28' S, 56º 50' W	1	E[a]	JX440495
Ar, E. Ríos, Colon	Co	32º 13' S, 58º 08' W	1	B[a]	JX440492
AR, Misiones, San Ignacio	SI	27º 15' S, 55º 32' W	1	G[a]	JX440497
BR, SC, Florianopolis	Fl	34º 18' S, 58º 57' W	1	H[a]	JX440491

a Extracted from Rodriguero *et al*. (2013)
