# Supplementary material for: Out of the forest: past and present range expansion of a parthenogenetic weevil pest, or how to colonize the world successfully
Source: Ecol Evol. 2016 Jul 6;6(15):5431–45. doi: 10.1002/ece3.2180 (PMC4984515; doi:10.1002/ece3.2180)
Supplement: Supplementary file 2 — Table S2. Georeferenced localities for Naupactus cervinus, including literature records, examined material from entomological collections and field sampling used for molecular studies and ecological niche modeling. [file ECE3-6-5431-s002.pdf]

Table S2 Georeferenced localities for *Naupactus cervinus*, including literature records, examined material from entomological collections and field sampling used for molecular studies and ecological niche modelling.
AR: Argentina, BR: Brazil, CH: Chile, PE: Peru, UR: Uruguay. Acronyms of entomological collections: CWOB: Charles O`Brien private collection, USA; FSCA: Florida State collection of arthropods, Gainesville, USA; IBUNAM: Instituto de Biología, Universidad Nacional Autónoma de México; MLP: Museo de La Plata collection, Argentina; TAMU: Texas A. & M. University, College Station, USA; URUC: Facultad de Ciencias, Universidad de la República collection, Uruguay.

Localities	***Latitude***	***Longitude***	***Reference***
AR, Buenos Aires, Buenos Aires	34° 36' S	58° 22' W	Field sampling
AR, Buenos Aires, Cardales	34° 18' S	58° 57' W	Field sampling
AR, Buenos Aires, La Plata	34° 55' S	57° 57' W	Lanteri AA (1986) Revisión del género *Asynonychus* Crotch (Coleoptera: Curculionidae). *Revista de la Asociación de Ciencias Naturales del Litoral*, **17**, 161-174.
AR, Buenos Aires, Los Acantilados	38° 07' S	57° 36' W	Lanteri AA (1986) Revisión del género *Asynonychus* Crotch (Coleoptera: Curculionidae). *Revista de la Asociación de Ciencias Naturales del Litoral*, **17**, 161-174.
AR, Buenos Aires, Magdalena	35° 04' S	57° 31' W	MLP
AR, Buenos Aires, Otamendi	34° 36' S	58° 25' W	Field sampling
AR, Buenos Aires, Pereyra Iraola	34° 50' S	58° 08' W	Field sampling
AR, Buenos Aires, Pergamino	33° 53' S	60° 34' W	Field sampling
AR, Buenos Aires, Punta Lara	34° 49' S	57° 59' W	MLP
AR, Buenos Aires, Talavera Island	34° 10' S	58° 30' W	Field sampling
AR, Buenos Aires, Tandil	37° 19' S	59° 08' W	Field sampling
AR, Buenos Aires, Tigre	34° 25' S	58° 34' W	Lanteri AA (1986) Revisión del género *Asynonychus* Crotch (Coleoptera: Curculionidae). *Revista de la Asociación de Ciencias Naturales del Litoral*, **17**, 161-174.
AR, Buenos Aires, Tres Lomas	36° 28' S	62° 52' W	Field sampling
AR, Buenos Aires, Zárate	34° 06' S	59° 01' W	Field sampling
AR, Catamarca	28° 28' S	65° 47' W	MLP

AR, Córdoba, Alta Gracia	31° 39' S	64° 25' W	Lanteri AA (1986) Revisión del género *Asynonychus* Crotch (Coleoptera: Curculionidae). *Revista de la Asociación de Ciencias Naturales del Litoral*, **17**, 161-174.
AR, Córdoba, Cabana	31° 13' S	64° 22' W	Lanteri AA (1986) Revisión del género *Asynonychus* Crotch (Coleoptera: Curculionidae). *Revista de la Asociación de Ciencias Naturales del Litoral*, **17**, 161-174.
AR, Córdoba, Champaquí	31° 59' S	64° 56' W	CWOB
AR, Córdoba, Cosquín	31° 14' S	64° 27' W	CWOB
AR, Córdoba, El Sauce	31° 06' S	64° 19' W	Lanteri AA (1986) Revisión del género *Asynonychus* Crotch (Coleoptera: Curculionidae). *Revista de la Asociación de Ciencias Naturales del Litoral*, **17**, 161-174.
AR, Córdoba, Huerta Grande	31° 04' S	64° 30' W	CWOB
AR, Córdoba, La Carlota	33° 25' S	63° 17' W	MLP
AR, Córdoba, La Cumbre	30° 59' S	64° 29' W	Lanteri AA (1986) Revisión del género *Asynonychus* Crotch (Coleoptera: Curculionidae). *Revista de la Asociación de Ciencias Naturales del Litoral*, **17**, 161-174.
AR, Córdoba, La Falda	31° 06' S	64° 30' W	Field sampling
AR, Córdoba, Los Cocos	30° 55' S	64° 29' W	Lanteri AA (1986) Revisión del género *Asynonychus* Crotch (Coleoptera: Curculionidae). *Revista de la Asociación de Ciencias Naturales del Litoral*, **17**, 161-174.
AR, Córdoba, Río Cuarto	33° 07' S	64° 20' W	Field sampling
AR, Córdoba, Tanti	31° 19' S	64° 35' W	Lanteri AA (1986) Revisión del género *Asynonychus* Crotch (Coleoptera: Curculionidae). *Revista de la Asociación de Ciencias Naturales del Litoral*, **17**, 161-174.
AR, Corrientes, Yapeyú	29° 28' S	56° 50' W	Field sampling
AR, Entre Ríos, Brazo Largo	33° 54' S	58° 53' W	Field sampling
AR, Entre Ríos, Chajarí	30° 47' S	57° 59' W	Field sampling
AR, Entre Ríos, El Palmar	31° 50' S	58° 17' W	Field sampling
AR, Entre Ríos, Gualeguaychú	33° 00' S	58° 31' W	Field sampling
AR, Entre Ríos, Salto Grande	30° 47' S	57° 55' W	Field sampling
AR, Jujuy, San Salvador de Jujuy	24° 11' S	65° 18' W	CWOB

AR, Jujuy, Yala	24° 07' S	65° 24' W	Lanteri AA (1986) Revisión del género *Asynonychus* Crotch (Coleoptera: Curculionidae). *Revista de la Asociación de Ciencias Naturales del Litoral*, **17**, 161-174.
AR, Mendoza, Chacras de Coria	32° 58' S	68° 52' W	Lanteri AA (1986) Revisión del género *Asynonychus* Crotch (Coleoptera: Curculionidae). *Revista de la Asociación de Ciencias Naturales del Litoral*, **17**, 161-174.
AR, Mendoza, Godoy Cruz	32° 55' S	68° 49' W	Field sampling
AR, Mendoza, Mendoza	33° 30' S	69° 00' W	Field sampling
AR, Misiones, Cerro Azul	27° 38' S	55° 30' W	Field sampling
AR, Misiones, Oberá	27° 29' S	55° 08' W	Field sampling
AR, Misiones, Pindapoy	27° 36' S	55° 49' W	Lanteri AA (1986) Revisión del género *Asynonychus* Crotch (Coleoptera: Curculionidae). *Revista de la Asociación de Ciencias Naturales del Litoral*, **17**, 161-174.
AR, Misiones, Puerto Londero	27° 22' S	54° 26' W	Lanteri AA (1986) Revisión del género *Asynonychus* Crotch (Coleoptera: Curculionidae). *Revista de la Asociación de Ciencias Naturales del Litoral*, **17**, 161-174.
AR, Misiones, Santa María	27° 54' S	55° 21' W	Lanteri AA (1986) Revisión del género *Asynonychus* Crotch (Coleoptera: Curculionidae). *Revista de la Asociación de Ciencias Naturales del Litoral*, **17**, 161-174.
AR, Salta, San Lorenzo	24° 44' S	65° 29' W	Lanteri AA (1986) Revisión del género *Asynonychus* Crotch (Coleoptera: Curculionidae). *Revista de la Asociación de Ciencias Naturales del Litoral*, **17**, 161-174.
AR, Salta, Río Piedras	25° 19' S	64° 55' W	MLP
AR, Santa Fe, Rosario	32° 57' S	60° 39' W	MLP
AR, Tucumán, San Miguel de Tucumán	26° 48' S	65° 13' W	MLP
AR, Tucumán, Siambón	26° 42' S	65° 27' W	Field sampling
BR, Minas Gerais, Passa Quatro	22° 23' S	44° 58' W	CWOB
BR, Paraná, Curitiba	25° 26' S	49° 16' W	CWOB
BR, Paraná, Laranjeiras do Sul	25° 24' S	52° 24' W	Field sampling
BR, Paraná, Ponta Grossa	25° 05' S	50° 09' W	Field sampling
BR, Paraná, Toledo	24° 42' S	53° 44' W	Field sampling

BR, Rio Grande do Sul, Alegrete	29° 46' S	55° 47' W	Field sampling
BR, Rio Grande do Sul, Bozano	28° 22' S	53° 46' W	Field sampling
BR, Rio Grande do Sul, Ijui	28° 23' S	53° 54' W	Field sampling
BR, Rio Grande do Sul, Itaára	29° 36' S	53° 45' W	Field sampling
BR, Rio Grande do Sul, Jari	29° 17' S	57° 13' W	Field sampling
BR, Rio Grande do Sul, Pelotas	31° 46' S	52° 21' W	CWOB
BR, Rio Grande do Sul, Porto Alegre	30° 02' S	51° 14' W	Chadwick C (1965) A review of Fuller's rose weevil *Pantomorus cervinus* (Boh.) (Col. Curculionidae). *Journal of the Entomological Society of Australia*, **2,** 1-11.
BR, Rio Grande do Sul, Santa María	29° 41' S	53° 48' W	Field sampling
BR, Rio Grande do Sul, São Sepé	30° 30' S	53° 28' W	Field sampling
BR, Santa Catarina, Nova Teutonia	27° 03' S	52° 24' W	Lanteri AA (1986) Revisión del género *Asynonychus* Crotch (Coleoptera: Curculionidae). *Revista de la Asociación de Ciencias Naturales del Litoral*, **17**, 161-174.
BR, Santa Catarina, Chapecó	27° 06' S	52° 37' W	MLP
BR, Sao Paulo, Américo Brasiliense	21° 43' S	48° 06' W	Guedes CJ, Lanteri AA & Parra JRP (2005) Chave de Identificação, Ocorrência e Distribuição dos Curculionídeos-das-raízes dos Citros em São Paulo e Minas Gerais. *Neotropical Entomology*, **34**, 577-584.
BR, Sao Paulo, Bebedouro	20° 57' S	48° 29' W	Guedes CJ, Lanteri AA & Parra JRP (2005) Chave de Identificação, Ocorrência e Distribuição dos Curculionídeos-das-raízes dos Citros em São Paulo e Minas Gerais. *Neotropical Entomology*, **34**, 577-584.
BR, Sao Paulo, Itapetininga	23° 36' S	48° 03' W	Guedes CJ, Lanteri AA & Parra JRP (2005) Chave de Identificação, Ocorrência e Distribuição dos Curculionídeos-das-raízes dos Citros em São Paulo e Minas Gerais. *Neotropical Entomology*, **34**, 577-584.
BR, Sao Paulo, Monte Azul Paulista	20° 54' S	48° 38' W	Guedes CJ, Lanteri AA & Parra JRP (2005) Chave de Identificação, Ocorrência e Distribuição dos Curculionídeos-das-raízes dos Citros em São Paulo e Minas Gerais. *Neotropical Entomology*, **34**, 577-584.
BR, Sao Paulo, Piracicaba	22° 43' S	47° 39' W	Guedes CJ, Lanteri AA & Parra JRP (2005) Chave de Identificação, Ocorrência e Distribuição dos Curculionídeos-das-raízes dos Citros em São Paulo e Minas

			Gerais. *Neotropical Entomology*, **34**, 577-584.
BR, Sao Paulo, San Paulo	23° 33' S	46° 38' W	CWOB
BR, Sao Paulo, Santa Rita do Passa Quatro	21° 44' S	47° 30' W	Guedes CJ, Lanteri AA & Parra JRP (2005) Chave de Identificação, Ocorrência e Distribuição dos Curculionídeos-das-raízes dos Citros em São Paulo e Minas Gerais. *Neotropical Entomology*, **34**, 577-584.
CH, Arauco	37° 05' S	73° 07' W	Elgueta M, Marvaldi A (2006) Lista sistemática de Curculionoidea presentes en Chile, con su sinonimia. *Boletín del Museo Nacional de Historia Natural de Chile*, **55**, 113-153.
CH, Arica	36° 58' S	72° 19' W	Elgueta M, Marvaldi A (2006) Lista sistemática de Curculionoidea presentes en Chile, con su sinonimia. *Boletín del Museo Nacional de Historia Natural de Chile*, **55**, 113-153.
CH, Cachapoal	34° 10' S	70° 37' W	Elgueta M, Marvaldi A (2006) Lista sistemática de Curculionoidea presentes en Chile, con su sinonimia. *Boletín del Museo Nacional de Historia Natural de Chile*, **55**, 113-153.
CH, Cardenal Caro	34° 17' S	71° 51' W	Elgueta M, Marvaldi A (2006) Lista sistemática de Curculionoidea presentes en Chile, con su sinonimia. *Boletín del Museo Nacional de Historia Natural de Chile*, **55**, 113-153.
CH, Choapa	31° 43' S	71° 13' W	Elgueta M, Marvaldi A (2006) Lista sistemática de Curculionoidea presentes en Chile, con su sinonimia. *Boletín del Museo Nacional de Historia Natural de Chile*, **55**, 113-153.
CH, Concepción	36° 48' S	73° 01' W	Elgueta M, Marvaldi A (2006) Lista sistemática de Curculionoidea presentes en Chile, con su sinonimia. *Boletín del Museo Nacional de Historia Natural de Chile*, **55**, 113-153.
CH, Copiapó	27° 22' S	70° 20' W	Elgueta M, Marvaldi A (2006) Lista sistemática de Curculionoidea presentes en Chile, con su sinonimia. *Boletín del Museo Nacional de Historia Natural de Chile*, **55**, 113-153.
CH, Coquimbo	29° 58' S	71° 20' W	Elgueta M, Marvaldi A (2006) Lista sistemática de Curculionoidea presentes en Chile, con su sinonimia. *Boletín del Museo Nacional de Historia Natural de Chile*, **55**, 113-153.

CH, Coordillera	33° 40' S	70° 09' W	Elgueta M, Marvaldi A (2006) Lista sistemática de Curculionoidea presentes en Chile, con su sinonimia. *Boletín del Museo Nacional de Historia Natural de Chile*, **55**, 113-153.
CH, Elqui	29° 49' S	70° 48' W	Lanteri AA (1986) Revisión del género *Asynonychus* Crotch (Coleoptera: Curculionidae). *Revista de la Asociación de Ciencias Naturales del Litoral*, **17**, 161-174.
CH, Huasco, Vallenar	28° 57' S	71° 15' W	Assayed for DNA
CH, Malleco	37° 48' S	72° 42' W	Elgueta M, Marvaldi A (2006) Lista sistemática de Curculionoidea presentes en Chile, con su sinonimia. *Boletín del Museo Nacional de Historia Natural de Chile*, **55**, 113-153.
CH, Ñuble	36° 43' S	71° 45' W	Elgueta M, Marvaldi A (2006) Lista sistemática de Curculionoidea presentes en Chile, con su sinonimia. *Boletín del Museo Nacional de Historia Natural de Chile*, **55**, 113-153.
CH, Ñuble, Chillán	36° 36' S	72° 07' W	Mander CV, Phillips CB, Glare TR, Chapman RB (2003) Preliminary assessment of COI and ITS1 sequence variation in Fuller's rose weevil. *New Zealand Plant Protection*, **56,** 190-193.
CH, Osorno	40° 34' S	73° 07' W	Elgueta M, Marvaldi A (2006) Lista sistemática de Curculionoidea presentes en Chile, con su sinonimia. *Boletín del Museo Nacional de Historia Natural de Chile*, **55**, 113-153.
CH, Quillota	32° 52' S	71° 14' W	Elgueta M, Marvaldi A (2006) Lista sistemática de Curculionoidea presentes en Chile, con su sinonimia. *Boletín del Museo Nacional de Historia Natural de Chile*, **55**, 113-153.
CH, Santiago	33° 26' S	70° 39' W	Assayed for DNA
CH, Valdivia	39° 49' S	73° 13' W	Elgueta M, Marvaldi A (2006) Lista sistemática de Curculionoidea presentes en Chile, con su sinonimia. *Boletín del Museo Nacional de Historia Natural de Chile*, **55**, 113-153.
CH, Valparaíso	33° 02' S	71° 37' W	Field sampling
CH, Valparaíso, Viña del Mar	33° 00' S	71° 31' W	Chadwick C (1965) A review of Fuller's rose weevil *Pantomorus cervinus* (Boh.) (Col. Curculionidae). *Journal of the Entomological Society of Australia*, **2,** 1-11.
CH, Juan Fernández Island	33° 38' S	78° 49' W	Elgueta M, Marvaldi A (2006) Lista sistemática de Curculionoidea presentes en Chile, con su sinonimia. *Boletín del Museo Nacional de Historia Natural de Chile*, **55,** 113-153.

PE, Tacna, Tacna	18° 01' S	70° 15' W	MLP
UR, Artigas, Arroyo Tres Cruces	30° 37' S	56° 37' W	URUC
UR, Canelones	34° 31' S	56° 16' W	Lanteri AA (1986) Revisión del género *Asynonychus* Crotch (Coleoptera: Curculionidae). *Revista de la Asociación de Ciencias Naturales del Litoral*, **17**, 161-174.
UR, Colonia, Paso de la Horqueta	34° 12' S	57° 52' W	CWOB
UR, Durazno, Paso de la Cruz	33° 56' S	56° 14' W	CWOB
UR, Maldonado, Cerro Pan de Azúcar	34° 47' S	55° 13' W	URUC
UR, Montevideo, Montevideo	34° 53' S	56° 11' W	Lanteri AA (1986) Revisión del género *Asynonychus* Crotch (Coleoptera: Curculionidae). *Revista de la Asociación de Ciencias Naturales del Litoral*, **17**, 161-174.
UR, Montevideo, Pocitos	34° 55' S	56° 09' W	URUC
UR, Montevideo, Sayago	34° 50' S	56° 13' W	CWOB
UR, Río Negro, Fray Bentos	33° 08' S	58° 18' W	CWOB
UR, Treinta y Tres, Treinta y Tres	33° 14' S	54° 22' W	CWOB
