# Supplementary material for: Out of the forest: past and present range expansion of a parthenogenetic weevil pest, or how to colonize the world successfully
Source: Ecol Evol. 2016 Jul 6;6(15):5431–45. doi: 10.1002/ece3.2180 (PMC4984515; doi:10.1002/ece3.2180)
Supplement: Supplementary file 3 — Table S3. Georeferenced localities for Naupactus cervinus, including literature records, examined material from entomological collections and field sampling used for molecular studies and ecological niche modeling. [file ECE3-6-5431-s003.pdf]

Table S3 Georeferenced localities for *Naupactus cervinus*, including literature records, examined material from entomological collections and field sampling used for molecular studies and ecological niche modelling.

AF: Africa, AI: Atlantic Islands, AS: Asia, AU: Australia, EU: Europe, NA: North America, NZ: New Zealand, PI, Pacific Islands.

Acronyms of entomological collections: FSCA: Florida State collection of arthropods, Gainesville, USA; IBUNAM: Instituto de Biología, Universidad Nacional Autónoma de México; TAMU: Texas A. & M. University, College Station, USA.

Localities	***Latitude***	***Longitude***	***Reference***
AF, Eritrea, Asmara	15° 20' N	38° 56' E	Chadwick C (1965) A review of Fuller's rose weevil *Pantomorus cervinus* (Boh.) (Col. Curculionidae). *Journal of the Entomological Society of Australia*, **2**, 1-11.
AF, Morocco, Oujda	34° 41' N	01° 55' W	Chadwick C (1965) A review of Fuller's rose weevil *Pantomorus cervinus* (Boh.) (Col. Curculionidae). *Journal of the Entomological Society of Australia*, **2**, 1-11.
AF, Morocco, Rabat	34° 01' N	06° 50' W	Chadwick C (1965) A review of Fuller's rose weevil *Pantomorus cervinus* (Boh.) (Col. Curculionidae). *Journal of the Entomological Society of Australia*, **2**, 1-11.
AF, South Africa, Cape Town	33° 55' S	18° 25' E	Chadwick C (1965) A review of Fuller's rose weevil *Pantomorus cervinus* (Boh.) (Col. Curculionidae). *Journal of the Entomological Society of Australia*, **2**, 1-11.
AF, South Africa, Johannesburg	26° 12' S	28° 03' E	Chadwick C (1965) A review of Fuller's rose weevil *Pantomorus cervinus* (Boh.) (Col. Curculionidae). *Journal of the Entomological Society of Australia*, **2**, 1-11.
AF, South Africa, Harrismith	28° 17' S	29° 08' E	Chadwick C (1965) A review of Fuller's rose weevil *Pantomorus cervinus* (Boh.) (Col. Curculionidae). *Journal of the Entomological Society of Australia*, **2**, 1-11.
AF, South Africa, King Williams Town	32° 53' S	27° 24' E	Chadwick C (1965) A review of Fuller's rose weevil *Pantomorus cervinus* (Boh.) (Col. Curculionidae). *Journal of the Entomological Society of Australia*, **2**, 1-11.
AF, South Africa, Ladysmith	28° 34' S	29° 47' E	Chadwick C (1965) A review of Fuller's rose weevil *Pantomorus cervinus* (Boh.) (Col. Curculionidae). *Journal of the Entomological Society of Australia*, **2**, 1-11.

AF, South Africa, Mountain Home	30° 15' S	29° 50' E	Chadwick C (1965) A review of Fuller's rose weevil *Pantomorus cervinus* (Boh.) (Col. Curculionidae). *Journal of the Entomological Society of Australia*, **2**, 1-11.
AF, South Africa, Muden	28° 58' S	30° 23' E	Chadwick C (1965) A review of Fuller's rose weevil *Pantomorus cervinus* (Boh.) (Col. Curculionidae). *Journal of the Entomological Society of Australia*, **2**, 1-11.
AF, South Africa, Pretoria	25° 44' S	28° 13' E	Chadwick C (1965) A review of Fuller's rose weevil *Pantomorus cervinus* (Boh.) (Col. Curculionidae). *Journal of the Entomological Society of Australia*, **2**, 1-11.
AF, South Africa, Richmond	29° 52' S	30° 17' E	Chadwick C (1965) A review of Fuller's rose weevil *Pantomorus cervinus* (Boh.) (Col. Curculionidae). *Journal of the Entomological Society of Australia*, **2**, 1-11.
AF, South Africa, Rustenburg	25° 40' S	27° 15' E	Chadwick C (1965) A review of Fuller's rose weevil *Pantomorus cervinus* (Boh.) (Col. Curculionidae). *Journal of the Entomological Society of Australia*, **2**, 1-11.
AI, Azores Islands, Fayal Island	38° 35' N	28° 42' W	Chadwick C (1965) A review of Fuller's rose weevil *Pantomorus cervinus* (Boh.) (Col. Curculionidae). *Journal of the Entomological Society of Australia*, **2**, 1-11.
AI, Madeira Islands, Madeira Island	32° 46' N	16° 58' W	Chadwick C (1965) A review of Fuller's rose weevil *Pantomorus cervinus* (Boh.) (Col. Curculionidae). *Journal of the Entomological Society of Australia*, **2**, 1-11.
AI, Canary Islands, Grand Canarie	27° 55' N	15° 33' W	Chadwick C (1965) A review of Fuller's rose weevil *Pantomorus cervinus* (Boh.) (Col. Curculionidae). *Journal of the Entomological Society of Australia*, **2**, 1-11.
AI, Canary Islands, Tenerife	28° 12' N	16° 36' W	Assayed for DNA
AI, Saint Helena Island	15° 58' S	05° 42' W	Chadwick C (1965) A review of Fuller's rose weevil *Pantomorus cervinus* (Boh.) (Col. Curculionidae). *Journal of the Entomological Society of Australia*, **2**, 1-11.
AI, Tristan da Cunha Island	37° 06' S	12° 17' W	Chadwick C (1965) A review of Fuller's rose weevil *Pantomorus cervinus* (Boh.) (Col. Curculionidae). *Journal of the Entomological Society of Australia*, **2**, 1-11.
AS, Israel	31° 46' N	35° 12' E	Friedman ALL(2009) Review of the biodiversity and zoogeographical patterns of th weevils (Coleoptera, Curculionoidea) in Israel. *ZooKeys*, **31**, 133-148.
AU, New South Wales, Albury	36° 05' S	146° 55' E	Chadwick C (1965) A review of Fuller's rose weevil *Pantomorus cervinus* (Boh.) (Col. Curculionidae). *Journal of the Entomological Society of Australia*, 2, 1-11.
AU, New South Wales, Armidale	30° 31' S	151° 40' E	Chadwick C. (1965) A review of Fuller's rose weevil *Pantomorus cervinus* (Boh.) (Col. Curculionidae). *Journal of the Entomological Society of Australia*, **2**, 1-11.

AU, New South Wales, Berowra	33° 37' S	151° 09' E	Chadwick C (1965) A review of Fuller's rose weevil *Pantomorus cervinus* (Boh.) (Col. Curculionidae). *Journal of the Entomological Society of Australia*, **2**, 1-11.
AU, New South Wales, Berry	34° 47' S	150° 42' E	Chadwick C (1965) A review of Fuller's rose weevil *Pantomorus cervinus* (Boh.) (Col. Curculionidae). *Journal of the Entomological Society of Australia*, **2**, 1-11.
AU, New South Wales, Blackheath	33° 38' S	150° 17' E	Chadwick C (1965) A review of Fuller's rose weevil *Pantomorus cervinus* (Boh.) (Col. Curculionidae). *Journal of the Entomological Society of Australia*, **2**, 1-11.
AU, New South Wales, Bulga	32° 39' S	151° 01' E	Chadwick C (1965) A review of Fuller's rose weevil *Pantomorus cervinus* (Boh.) (Col. Curculionidae). *Journal of the Entomological Society of Australia*, **2**, 1-11.
AU, New South Wales, Coomealla	34° 05' S	142° 04' E	Chadwick C (1965) A review of Fuller's rose weevil *Pantomorus cervinus* (Boh.) (Col. Curculionidae). *Journal of the Entomological Society of Australia*, **2**, 1-11.
AU, New South Wales, Coonabarabran	31° 16' S	149° 17' E	Chadwick C (1965) A review of Fuller's rose weevil *Pantomorus cervinus* (Boh.) (Col. Curculionidae). *Journal of the Entomological Society of Australia*, **2**, 1-11.
AU, New South Wales, Cowra	33° 50' S	148° 41' E	Chadwick C (1965) A review of Fuller's rose weevil *Pantomorus cervinus* (Boh.) (Col. Curculionidae). *Journal of the Entomological Society of Australia*, **2**, 1-11.
AU, New South Wales, Cremorne	33° 50' S	151° 14' E	Chadwick C (1965) A review of Fuller's rose weevil *Pantomorus cervinus* (Boh.) (Col. Curculionidae). *Journal of the Entomological Society of Australia*, **2**, 1-11.
AU, New South Wales, Dapto	34° 30' S	150° 48' E	Chadwick C (1965) A review of Fuller's rose weevil *Pantomorus cervinus* (Boh.) (Col. Curculionidae). *Journal of the Entomological Society of Australia*, **2**, 1-11.
AU, New South Wales, Deniliquin	35° 31' S	144° 59' E	Chadwick C (1965) A review of Fuller's rose weevil *Pantomorus cervinus* (Boh.) (Col. Curculionidae). *Journal of the Entomological Society of Australia*, **2**, 1-11.
AU, New South Wales, Gosford	33° 26' S	151° 21' E	Chadwick C (1965) A review of Fuller's rose weevil *Pantomorus cervinus* (Boh.) (Col. Curculionidae). *Journal of the Entomological Society of Australia*, **2**, 1-11.
AU, New South Wales, Graman	29° 28' S	150° 56' E	Chadwick C (1965) A review of Fuller's rose weevil *Pantomorus cervinus* (Boh.) (Col. Curculionidae). *Journal of the Entomological Society of Australia*, **2**, 1-11.
AU, New South Wales, Griffith	34° 17' S	146° 03' E	Chadwick C 1965) A review of Fuller's rose weevil *Pantomorus cervinus* (Boh.) (Co[illegible] Curculionidae). *Journal of the Entomological Society of Australia*, **2**, 1-11.
AU, New South Wales, Inverell	29° 47' S	151° 07' E	Chadwick C (1965) A review of Fuller's rose weevil *Pantomorus cervinus* (Boh.) (Col. Curculionidae). *Journal of the Entomological Society of Australia*, **2**, 1-11.
AU, New South Wales, Kempsey	31° 05' S	152° 50' E	Chadwick C (1965) A review of Fuller's rose weevil *Pantomorus cervinus* (Boh.) (Col. Curculionidae). *Journal of the Entomological Society of Australia*, **2**, 1-11.
AU, New South Wales, Kincumber	33° 28' S	151° 23' E	Chadwick C (1965) A review of Fuller's rose weevil *Pantomorus cervinus* (Boh.) (Col. Curculionidae). *Journal of the Entomological Society of Australia*, **2**, 1-11.

AU, New South Wales, Kurrajong Heights	33° 32' S	150° 38' E	Chadwick C (1965) A review of Fuller's rose weevil *Pantomorus cervinus* (Boh.) (Col. Curculionidae). *Journal of the Entomological Society of Australia*, **2**, 1-11.
AU, New South Wales, Lismore	28° 49' S	153° 17' E	Chadwick C (1965) A review of Fuller's rose weevil *Pantomorus cervinus* (Boh.) (Col. Curculionidae). *Journal of the Entomological Society of Australia*, **2**, 1-11.
AU, New South Wales, Leeton	34° 33' S	146° 24' E	Chadwick C (1965) A review of Fuller's rose weevil *Pantomorus cervinus* (Boh.) (Col. Curculionidae). *Journal of the Entomological Society of Australia*, **2**, 1-11.
AU, New South Wales, Matcham	33° 25' S	151° 25' E	Chadwick C (1965) A review of Fuller's rose weevil *Pantomorus cervinus* (Boh.) (Col. Curculionidae). *Journal of the Entomological Society of Australia*, **2**, 1-11.
AU, New South Wales, Mirool	34° 19' S	147° 05' E	Chadwick C (1965) A review of Fuller's rose weevil *Pantomorus cervinus* (Boh.) (Col. Curculionidae). *Journal of the Entomological Society of Australia*, **2**, 1-11.
AU, New South Wales, Murwillumbah	28° 20' S	153° 24' E	Chadwick C (1965) A review of Fuller's rose weevil *Pantomorus cervinus* (Boh.) (Col. Curculionidae). *Journal of the Entomological Society of Australia*, **2**, 1-11.
AU, New South Wales, Nambucca	30° 39' S	153° 00' E	Chadwick C (1965) A review of Fuller's rose weevil *Pantomorus cervinus* (Boh.) (Col. Curculionidae). *Journal of the Entomological Society of Australia*, **2**, 1-11.
AU, New South Wales, Nashdale	33° 18' S	149° 01' E	Chadwick C (1965) A review of Fuller's rose weevil *Pantomorus cervinus* (Boh.) (Col. Curculionidae). *Journal of the Entomological Society of Australia*, **2**, 1-11.
AU, New South Wales, Ourimbah	33° 22' S	151° 22' E	Chadwick C (1965) A review of Fuller's rose weevil *Pantomorus cervinus* (Boh.) (Col. Curculionidae). *Journal of the Entomological Society of Australia*, **2**, 1-11.
AU, New South Wales, Port Macquarie	31° 26' S	152° 54' E	Chadwick C (1965) A review of Fuller's rose weevil *Pantomorus cervinus* (Boh.) (Col. Curculionidae). *Journal of the Entomological Society of Australia*, **2**, 1-11.
AU, New South Wales, Sydney	33° 52' S	151° 12' E	Chadwick C (1965) A review of Fuller's rose weevil *Pantomorus cervinus* (Boh.) (Col. Curculionidae). *Journal of the Entomological Society of Australia*, **2**, 1-11.
AU, New South Wales, Tamworth	31° 05' S	150° 56' E	Chadwick C (1965) A review of Fuller's rose weevil *Pantomorus cervinus* (Boh.) (Col. Curculionidae). *Journal of the Entomological Society of Australia*, **2**, 1-11.
AU, New South Wales, Taree	31° 54' S	152° 27' E	Chadwick C (1965) A review of Fuller's rose weevil *Pantomorus cervinus* (Boh.) (Col. Curculionidae). *Journal of the Entomological Society of Australia*, **2**, 1-11.
AU, New South Wales, Yenda	34° 15' S	146° 12' E	Chadwick C (1965) A review of Fuller's rose weevil *Pantomorus cervinus* (Boh.) (Col. Curculionidae). *Journal of the Entomological Society of Australia*, **2**, 1-11.
AU, Queensland	20° 55' S	142° 42' E	Chadwick C (1965) A review of Fuller's rose weevil *Pantomorus cervinus* (Boh.) (Col. Curculionidae). *Journal of the Entomological Society of Australia*, **2**, 1-11.
AU, Victoria, Canberra	35° 18' S	149° 07' E	Chadwick C (1965) A review of Fuller's rose weevil *Pantomorus cervinus* (Boh.) (Col. Curculionidae). *Journal of the Entomological Society of Australia*, **2**, 1-11.

AU, Victoria, Cheltenham	37° 58' S	145° 03' E	Mander, C.V., Phillips, C.B., Glare, T.R. & Chapman, R.B. (2003) Preliminary assessment of COI and ITS1 sequence variation in Fuller's rose weevil. *New* Zealand *Plant Protection*, 56, 190
AU, Victoria, Tatura	36° 26' S	145° 14' E	Mander, C.V., Phillips, C.B., Glare, T.R. & Chapman, R.B. (2003) Preliminary assessment of COI and ITS1 sequence variation in Fuller's rose weevil. *New* Zealand *Plant Protection*, 56, 190
AU, Victoria, Vermont	37° 50' S	145° 12' E	Chadwick, C. (1965) A review of Fuller's rose weevil *Pantomorus cervinus* (Boh.) (Col. Curculionidae). *Journal of the Entomological Society of Australia*, **2**, 1-11.
AU, Western Australia, Carmel	32° 01' S	116° 06' E	Chadwick, C. (1965) A review of Fuller's rose weevil *Pantomorus cervinus* (Boh.) (Col. Curculionidae). *Journal of the Entomological Society of Australia*, **2**, 1-11.
AU, Western Australia, Denmark	34° 58' S	117° 21' E	Chadwick, C. (1965) A review of Fuller's rose weevil *Pantomorus cervinus* (Boh.) (Col. Curculionidae). *Journal of the Entomological Society of Australia*, **2**, 1-11.
AU, Western Australia, Pickering	27° 53' S	121° 17' E	Chadwick, C. (1965) A review of Fuller's rose weevil *Pantomorus cervinus* (Boh.) (Col. Curculionidae). *Journal of the Entomological Society of Australia*, **2**, 1-11.
AU, Lord Howe Island	31° 33' S	159° 05' E	Chadwick, C. (1965) A review of Fuller's rose weevil *Pantomorus cervinus* (Boh.) (Col. Curculionidae). *Journal of the Entomological Society of Australia*, **2**, 1-11.
AU, Norfolk Island	29° 02' S	167° 57' E	Chadwick, C. (1965) A review of Fuller's rose weevil *Pantomorus cervinus* (Boh.) (Col. Curculionidae). *Journal of the Entomological Society of Australia*, **2**, 1-11.
NZ, Auckland, Awhitu	37° 05' S	174° 38' E	Mander CV, Phillips CB, Glare TR, Chapman RB (2003) Preliminary assessment of COI and ITS1 sequence variation in Fuller's rose weevil. *New Zealand Plant Protection*, **56**, 190-193.
NZ, Auckland	36° 50' S	174° 45' E	Chadwick C (1965) A review of Fuller's rose weevil Pantomorus cervinus (Boh.) (Col. Curculionidae). *Journal of the Entomological Society of Australia*, **2**, 1-11.
NZ, Nelson	41° 16' S	173° 17' E	Chadwick C (1965) A review of Fuller's rose weevil *Pantomorus cervinus* (Boh.) (Col. Curculionidae). *Journal of the Entomological Society of Australia*, **2**, 1-11.
NZ, North Island, Bay of Plenty	38° 05' S	176° 25' E	Mander CV, Phillips CB, Glare TR, Chapman RB (2003) Preliminary assessment of COI and ITS1 sequence variation in Fuller's rose weevil. *New Zealand Plant Protection*, **56**, 190-193.
PI, Easter Island	27° 06' S	109° 21' E	Assayed for DNA
PI, French Polynesia, Tahiti	17° 39' S	149° 25' E	Assayed for DNA

PI, French Polynesia, Rapa Island	27° 35' S	144° 35' E	Assayed for DNA
PI, Hawaii, Big Island	19º 36’ N,	155º 39’ W	Assayed for DNA
PI, Hawaii, Kauai	22º 07’ N	159º 31’ W	Assayed for DNA
PI, Hawaii, Oahu	21° 26' N	158° 00' W	Buchanan LL (1939) The species of *Pantomorus* of America north of Mexico. *U.S. Department of Agriculture Miscellaneous Publications*, **341**, 1-39.
PI, Hawaii, Maui	20° 48' N	156° 20' W	Buchanan LL (1939) The species of *Pantomorus* of America north of Mexico. *U.S. Department of Agriculture Miscellaneous Publications*, **341**, 1-39.
EU, France, Bouches-du-Rhone	43° 35' N	05° 19' E	Chadwick C (1965) A review of Fuller's rose weevil *Pantomorus cervinus* (Boh.) (Col. Curculionidae). *Journal of the Entomological Society of Australia*, **2**, 1-11.
EU, France, Gironde	44° 51' N	00° 27' W	Chadwick C (1965) A review of Fuller's rose weevil *Pantomorus cervinus* (Boh.) (Col. Curculionidae). *Journal of the Entomological Society of Australia*, **2**, 1-11.
EU, France, Le Havre	49° 30' N	00° 06' E	Chadwick C (1965) A review of Fuller's rose weevil *Pantomorus cervinus* (Boh.) (Col. Curculionidae). *Journal of the Entomological Society of Australia*, **2**, 1-11.
EU, France, Lyonnais	47º 39' N	02º 10' E	Chadwick C (1965) A review of Fuller's rose weevil *Pantomorus cervinus* (Boh.) (Col. Curculionidae). *Journal of the Entomological Society of Australia*, **2**, 1-11.
EU, France, Marseilles	43° 18' N	05° 22' E	Chadwick C (1965) A review of Fuller's rose weevil *Pantomorus cervinus* (Boh.) (Col. Curculionidae). *Journal of the Entomological Society of Australia*, **2**, 1-11.
EU, France, Eastern Pyrenees	42° 36' N	02° 32' E	Chadwick C (1965) A review of Fuller's rose weevil *Pantomorus cervinus* (Boh.) (Col. Curculionidae). *Journal of the Entomological Society of Australia*, **2**, 1-11.
EU, France, Toulon	43° 07' N	05° 56' E	Chadwick C (1965) A review of Fuller's rose weevil *Pantomorus cervinus* (Boh.) (Col. Curculionidae). *Journal of the Entomological Society of Australia*, **2**, 1-11.
EU, Italy, Liguria	44° 25' N	08° 32' E	Chadwick C (1965) A review of Fuller's rose weevil *Pantomorus cervinus* (Boh.) (Col. Curculionidae). *Journal of the Entomological Society of Australia*, **2**, 1-11.
EU, Italy, Sicily, Palermo	38° 07' N	13° 22' E	Chadwick C (1965) A review of Fuller's rose weevil *Pantomorus cervinus* (Boh.) (Col. Curculionidae). *Journal of the Entomological Society of Australia*, **2**, 1-11.
EU, Malta	35° 56' N	14° 25' E	Mifsud D, Colonelli E (2010) The Curculionoidea of the Maltese Islands (Central Mediterranean) (Coleoptera). *Bulletin of the Entomological Society of Malt*[illegible] **3**, 55-143.
EU, Spain, Valencia	39° 29' N	00° 23' W	Assayed for DNA

NA, Canada, Brantford	43° 09' N	80° 16' W	Chadwick C (1965) A review of Fuller's rose weevil *Pantomorus cervinus* (Boh.) (Col. Curculionidae). *Journal of the Entomological Society of Australia*, **2**, 1-11.
NA Canada, Ontario	51° 11' N	87° 20' W	Bright DE, Bouchard P (2008) *The insects and arachnids Canada. Part 25. Coleoptera, Curculionidae, Entiminae*. NRC Research Press, Ottawa and Ontario.
NA, Canada, Ottawa	45° 25' N	75° 42' W	Chadwick C (1965) A review of Fuller's rose weevil *Pantomorus cervinus* (Boh.) (Col. Curculionidae). *Journal of the Entomological Society of Australia*, **2**, 1-11.
NA, Canada, Stewarton	45° 43' N	65° 49' W	Chadwick C (1965) A review of Fuller's rose weevil *Pantomorus cervinus* (Boh.) (Col. Curculionidae). *Journal of the Entomological Society of Australia*, **2**, 1-11.
NA, Mexico, Federal District, Chapultepec	19° 12' N	99° 34' W	IBUNAM
NA, Mexico, Guanajuato, Guanajuato	21° 01' N	101° 15' W	Buchanan LL (1939) The species of *Pantomorus* of America north of Mexico. *U.S. Department of Agriculture Miscellaneous Publications*, **341**, 1-39.
NA, Mexico, Querétaro, Tequisquiapan	20° 31' N	99° 53' W	IBUNAM
NA, USA, Alabama, Montgomery	32° 22' N	86° 18' W	FSCA
NA, USA, Arizona	34° 03' N	111° 06' W	Chadwick C (1965) A review of Fuller's rose weevil *Pantomorus cervinus* (Boh.) (Col. Curculionidae). *Journal of the Entomological Society of Australia*, **2**, 1-11.
NA, USA, California, Los Angeles	34° 03' N	118° 15' W	Chadwick C. (1965) A review of Fuller's rose weevil *Pantomorus cervinus* (Boh.) (Col. Curculionidae). *Journal of the Entomological Society of Australia*, **2**, 1-11.
NA, USA, California, San Diego	32° 43' N	117° 09' W	Chadwick C (1965) A review of Fuller's rose weevil *Pantomorus cervinus* (Boh.) (Col. Curculionidae). *Journal of the Entomological Society of Australia*, **2**, 1-11.
NA, USA, California, Sta. Cruz County	36° 58' N	122° 02' W	TAMU
NA, USA, Connecticut	41° 36' N	73° 05' W	Chadwick C (1965) A review of Fuller's rose weevil *Pantomorus cervinus* (Boh.) (Col. Curculionidae). *Journal of the Entomological Society of Australia*, **2**, 1-11.
NA, USA, Columbia district	38° 54' N	77° 02' W	Chadwick C (1965) A review of Fuller's rose weevil *Pantomorus cervinus* (Boh.) (Col. Curculionidae). *Journal of the Entomological Society of Australia*, **2**, 1-11.
NA, USA, Florida, Jackson County	30° 43' N	85° 11' W	FSCA
NA, USA, Florida, Lake City	30° 11' N	82° 38' W	FSCA
NA, USA, Florida, Fort Meade	27° 45' N	81° 48' W	FSCA
NA, USA, Florida, Pensacola	30° 25' N	87° 13' W	FSCA
NA, USA, Florida, Oneco	27° 27' N	82° 33' W	FSCA
NA, USA, Florida, Quincy	30° 35' N	84° 35' W	FSCA

NA, USA, Georgia	32° 09' N	82° 54' W	Chadwick C (1965) A review of Fuller's rose weevil *Pantomorus cervinus* (Boh.) (Col. Curculionidae). *Journal of the Entomological Society of Australia*, **2**, 1-11.
NA, USA, Illinois	40° 38' N	89° 24' W	Chadwick C (1965) A review of Fuller's rose weevil *Pantomorus cervinus* (Boh.) (Col. Curculionidae). *Journal of the Entomological Society of Australia*, **2**, 1-11.
NA, USA, Indiana	40° 33' N	85° 36' W	Chadwick C (1965) A review of Fuller's rose weevil *Pantomorus cervinus* (Boh.) (Col. Curculionidae). *Journal of the Entomological Society of Australia*, **2**, 1-11.
NA, USA, Iowa	43° 01' N	93° 19' W	Chadwick C (1965) A review of Fuller's rose weevil *Pantomorus cervinus* (Boh.) (Col. Curculionidae). *Journal of the Entomological Society of Australia*, **2**, 1-11.
NA, USA, Louisiana	31° 15' N	92° 09' W	Chadwick C (1965) A review of Fuller's rose weevil *Pantomorus cervinus* (Boh.) (Col. Curculionidae). *Journal of the Entomological Society of Australia*, **2**, 1-11.
NA, USA, Maine	45° 20' N	68° 51' W	Chadwick C (1965) A review of Fuller's rose weevil *Pantomorus cervinus* (Boh.) (Col. Curculionidae). *Journal of the Entomological Society of Australia*, **2**, 1-11.
NA, USA, Maryland	39° 03' N	76° 38' W	Chadwick C (1965) A review of Fuller's rose weevil *Pantomorus cervinus* (Boh.) (Col. Curculionidae). *Journal of the Entomological Society of Australia*, **2**, 1-11.
NA, USA Massachusetts, Boston	42° 23' N	71° 04' W	Chadwick C (1965) A review of Fuller's rose weevil *Pantomorus cervinus* (Boh.) (Col. Curculionidae). *Journal of the Entomological Society of Australia*, **2**, 1-11.
NA, USA, Massachussets, Worcester	42° 16' N	71° 48' W	Chadwick C (1965) A review of Fuller's rose weevil *Pantomorus cervinus* (Boh.) (Col. Curculionidae). *Journal of the Entomological Society of Australia*, **2**, 1-11.
NA, USA, Michigan	44° 19' N	85° 36' W	Chadwick C (1965) A review of Fuller's rose weevil *Pantomorus cervinus* (Boh.) (Col. Curculionidae). *Journal of the Entomological Society of Australia*, **2**, 1-11.
NA, USA, Mississippi	33° 35' N	89° 30' W	Chadwick C (1965) A review of Fuller's rose weevil *Pantomorus cervinus* (Boh.) (Col. Curculionidae). *Journal of the Entomological Society of Australia*, **2**, 1-11.
NA, USA, Mississippi, Harrison County	30° 26' N	89° 01' W	TAMU
NA, USA, Missouri	37° 58' N	91° 50' W	Chadwick C (1965) A review of Fuller's rose weevil *Pantomorus cervinus* (Boh.) (Col. Curculionidae). *Journal of the Entomological Society of Australia*, **2**, 1-11.
NA, USA, Montana	46° 53' N	110° 22' W	Chadwick C (1965) A review of Fuller's rose weevil *Pantomorus cervinus* (Boh.) (Col. Curculionidae). *Journal of the Entomological Society of Australia*, **2**, 1-11.
NA, USA, Nebraska	41° 35' N	99° 26' W	Chadwick C (1965) A review of Fuller's rose weevil *Pantomorus cervinus* (Boh.) (Col. Curculionidae). *Journal of the Entomological Society of Australia*, **2**, 1-11.
NA, USA, New Jersey, Union County	40° 40' N	74° 17' W	Chadwick C (1965) A review of Fuller's rose weevil *Pantomorus cervinus* (Boh.) (Col. Curculionidae). *Journal of the Entomological Society of Australia*, **2**, 1-11.

NA, USA, New York, Albany	42° 51' N	73° 44' W	Chadwick C (1965) A review of Fuller's rose weevil *Pantomorus cervinus* (Boh.) (Col. Curculionidae). *Journal of the Entomological Society of Australia*, **2**, 1-11.
NA, USA, North Carolina	35° 46' N	79° 01' W	Chadwick C (1965) A review of Fuller's rose weevil *Pantomorus cervinus* (Boh.) (Col. Curculionidae). *Journal of the Entomological Society of Australia*, **2**, 1-11.
NA, USA, Ohio	40° 25' N	82° 54' W	Chadwick C (1965) A review of Fuller's rose weevil *Pantomorus cervinus* (Boh.) (Col. Curculionidae). *Journal of the Entomological Society of Australia*, **2**, 1-11.
NA, USA, Oklahoma	35° 00' N	97° 06' W	Chadwick C (1965) A review of Fuller's rose weevil *Pantomorus cervinus* (Boh.) (Col. Curculionidae). *Journal of the Entomological Society of Australia*, **2**, 1-11.
NA, USA, Oregon	43° 48' N	120° 33' W	Chadwick C (1965) A review of Fuller's rose weevil *Pantomorus cervinus* (Boh.) (Col. Curculionidae). *Journal of the Entomological Society of Australia*, **2**, 1-11.
NA, USA, Pennsylvania	33° 50' N	81° 09' W	Chadwick C (1965) A review of Fuller's rose weevil *Pantomorus cervinus* (Boh.) (Col. Curculionidae). *Journal of the Entomological Society of Australia*, **2**, 1-11.
NA, USA, South Carolina	33° 50' N	81° 10' W	Chadwick C (1965) A review of Fuller's rose weevil *Pantomorus cervinus* (Boh.) (Col. Curculionidae). *Journal of the Entomological Society of Australia*, **2**, 1-11.
NA, USA, Tennessee	35° 53' N	86° 24' W	Chadwick C (1965) A review of Fuller's rose weevil *Pantomorus cervinus* (Boh.) (Col. Curculionidae). *Journal of the Entomological Society of Australia*, **2**, 1-11.
NA, USA, Texas	29° 42' N	98° 36' W	Chadwick C (1965) A review of Fuller's rose weevil *Pantomorus cervinus* (Boh.) (Col. Curculionidae). *Journal of the Entomological Society of Australia*, **2**, 1-11.
NA, USA, Texas, Davis County	30° 35' N	103° 54' W	TAMU
NA, USA, Virginia	37° 26' N	78° 39' W	Chadwick C (1965) A review of Fuller's rose weevil *Pantomorus cervinus* (Boh.) (Col. Curculionidae). *Journal of the Entomological Society of Australia*, **2**, 1-11.
NA, USA, Wisconsin	43° 47' N	88° 47' W	Chadwick C (1965) A review of Fuller's rose weevil *Pantomorus cervinus* (Boh.) (Col. Curculionidae). *Journal of the Entomological Society of Australia*, **2**, 1-11.
